# Integrative transcriptome network analysis of iPSC-derived neurons from schizophrenia and schizoaffective disorder patients with 22q11.2 deletion

**DOI:** 10.1186/s12918-016-0366-0

**Published:** 2016-11-15

**Authors:** Mingyan Lin, Erika Pedrosa, Anastasia Hrabovsky, Jian Chen, Benjamin R. Puliafito, Stephanie R. Gilbert, Deyou Zheng, Herbert M. Lachman

**Affiliations:** 1 Department of Genetics, Albert Einstein College of Medicine, 1300 Morris Park Ave., Bronx, NY USA; 2 Department of Psychiatry and Behavioral Sciences, Albert Einstein College of Medicine, 1300 Morris Park Ave., Bronx, NY USA; 3 Department of Neurology, Albert Einstein College of Medicine, 1300 Morris Park Ave., Bronx, NY USA; 4 Department of Neuroscience, Albert Einstein College of Medicine, 1300 Morris Park Ave., Bronx, NY USA; 5 Department of Medicine, Albert Einstein College of Medicine, 1300 Morris Park Ave., Bronx, NY USA

## Abstract

**Background:**

Individuals with 22q11.2 Deletion Syndrome (22q11.2 DS) are a specific high-risk group for developing schizophrenia (SZ), schizoaffective disorder (SAD) and autism spectrum disorders (ASD). Several genes in the deleted region have been implicated in the development of SZ, e.g., *PRODH* and *DGCR8*. However, the mechanistic connection between these genes and the neuropsychiatric phenotype remains unclear. To elucidate the molecular consequences of 22q11.2 deletion in early neural development, we carried out RNA-seq analysis to investigate gene expression in early differentiating human neurons derived from induced pluripotent stem cells (iPSCs) of 22q11.2 DS SZ and SAD patients.

**Methods:**

Eight cases (ten iPSC-neuron samples in total including duplicate clones) and seven controls (nine in total including duplicate clones) were subjected to RNA sequencing. Using a systems level analysis, differentially expressed genes/gene-modules and pathway of interests were identified. Lastly, we related our findings from in vitro neuronal cultures to brain development by mapping differentially expressed genes to BrainSpan transcriptomes.

**Results:**

We observed ~2-fold reduction in expression of almost all genes in the 22q11.2 region in SZ (37 genes reached *p*-value < 0.05, 36 of which reached a false discovery rate < 0.05). Outside of the deleted region, 745 genes showed significant differences in expression between SZ and control neurons (*p* < 0.05). Function enrichment and network analysis of the differentially expressed genes uncovered converging evidence on abnormal expression in key functional pathways, such as apoptosis, cell cycle and survival, and MAPK signaling in the SZ and SAD samples. By leveraging transcriptome profiles of normal human brain tissues across human development into adulthood, we showed that the differentially expressed genes converge on a sub-network mediated by *CDC45* and the cell cycle, which would be disrupted by the 22q11.2 deletion during embryonic brain development, and another sub-network modulated by *PRODH*, which could contribute to disruption of brain function during adolescence.

**Conclusions:**

This study has provided evidence for disruption of potential molecular events in SZ patient with 22q11.2 deletion and related our findings from in vitro neuronal cultures to functional perturbations that can occur during brain development in SZ.

**Electronic supplementary material:**

The online version of this article (doi:10.1186/s12918-016-0366-0) contains supplementary material, which is available to authorized users.

## Background

SZ is a very complex disorder caused by multivariate genetic and environmental factors. Apart from familial factors, such as being the monozygotic co-twin of a SZ proband or being the offspring of two parents with the condition, 22q11.2 deletion is the highest known risk factor for the development of SZ [1]. 22q11.2 Deletion Syndrome (22q11.2DS) has a highly variable clinical presentation including velo-cardio-facial syndrome (VCFS), cognitive and behavioral disorders, and SZ-like psychosis [2–7]. Several genes in the deleted region have been suggested as candidates for the development of SZ, including *TBX1* [8], *COMT* [9–11], *GNB1L* [12], *PRODH* [13–16], and *DGCR8* [17–19]. It is, however, thought that the 22q11.2 DS reflects combinatorial effects of diminished dosage of multiple genes/miRNAs acting on common cellular processes involved in neuronal development and neurotransmission [20, 21]. From this perspective, it is speculated that downstream targets affected by deleted genes may be enriched in cellular pathways involved in neuronal development or neuronal activity, and that reduced expression of the deleted genes may dysregulate these pathways.

Thus, it is valuable to search for SZ-specific changes in early neural development of individuals containing the 22q11.2 deletion. However, research on the biological basis of SZ and other neuropsychiatric disorders has been hampered by the inaccessibility of developing human brains. This problem has been partially circumvented by iPSC technology [22], which allows investigators to grow patient-specific neurons or neuroaggregates [23, 24] for modeling in vitro the cellular developmental abnormalities associated with psychiatric disorders. In the past few years, investigators have successfully applied this strategy and established iPSC lines in a variety of brain disorders including Rett Syndrome, Parkinson Disease, Amyotrophic Lateral Sclerosis, Familial Dysautonomia, and most recently, SZ [25–30].

In this study, we performed a global and unbiased transcriptome analysis of iPSC-derived neurons from SZ and SAD patients with 22q11.2 deletion in comparison with neurons from healthy individuals (without the deletion). We reasoned that molecular changes would be easier to uncover from 22q11.2 deletion patients with SZ than other genetic subgroups, as 22q11.2 deletion is the most common known genetic risk factor and is associated with a very high penetrance, and the results could shed light on the molecular abnormalities and gene network disruption (due to combinatory effects of some 22q11.2 genes and candidates genes outside the region) in SZ developing brains. In addition to the two-fold reduction in the expression of genes that map to the 22q11.2 deleted region, our results showed altered expression of genes involved in apoptosis, cell cycle and survival, and MAPK signaling. These results are consistent with a number of previous reports showing abnormal apoptotic function in the neurodevelopmental and neurodegenerative processes associated with SZ [31–33]. Moreover, our analysis suggested that there might be an inter-chromosomal interaction between the 22q11.2 region and the *HLA* locus on 6p21, which points to a potential functional connection. Lastly, through mapping differentially expressed genes to the BrainSpan transcriptomes, we found that they converge on two networks of genes co-expressed in the embryonic stage and adolescence, with specific functional clusters critical to neurodevelopment and neuronal functions. Overall, our results indicate that early differentiating neurons derived from iPSCs with 22q11.2 deletions provide a model for studying SZ-related phenomena and uncovering neurodevelopmental disruptions, which could potentially be generalized to the other genetic subgroups.

## Methods

### Development of iPSCs from skin fibroblasts

Controls and patients with 22q11.2 del diagnosed with a psychotic disorder (SZ, childhood onset schizophrenia [COS], SAD) were recruited from two settings, the Albert Einstein College of Medicine (AECOM) and the National Institutes of Mental Health (NIMH), Child Psychiatry Branch. For simplicity, we will usually refer to the patient samples as SZ. The study and consent forms for the AECOM cohort were approved by the AECOM Institution Review Board (IRB) and were signed by the subjects at a time when psychotic symptoms were well-controlled with medications. For the NIMH subjects, the study and consent were approved by the NIMH IRB. For children, consent was obtained from parents, and assent was obtained from participants. Subjects were not disadvantaged in any way if they refused to participate in the study. Consent was obtained by skilled members of the research teams who had received prior human subjects training. All patients have confirmed 22q11.2 deletion as determined by FISH or CGH arrays [34]. A summary of the patients and controls used in this study are shown in Additional file 1, and more detailed clinical descriptions are provided in Additional file 2.

The iPSC lines were generated from fibroblasts obtained from skin biopsies performed by board-certified physicians. The procedure for growing fibroblasts in preparation for reprogramming into iPSCs is detailed in Additional file 2. Briefly, iPSC reprogramming was carried out by nucleofection. One vial of fibroblasts was thawed out and placed in a T75 flask in DMEM/F12 supplemented with 10% FBS and fed every 2 days. Cells were grown to ~50% confluence (~4–5 days), after which they were trypsinized and subjected to nucleofection (~6 x10^5^ cells). Reprogramming was carried out using an Amaxa 4D-Nucleofector (P2 Primary Cell Kit from Lonza catalog# V4XP-2012, Program FF-135) with non-integrating plasmids containing *OCT4, SOX2, KLF4, L-MYC, LIN28,* and a p53 shRNA vector (Addgene Cat. # 27077, 27078, 27080), according to Okita et al., with some modifications [24, 35, 36]. iPSCs were maintained on Matrigel plates in mTeSR1 medium (Stem Cell Technologies) with daily feeding in 37 °C/5% CO_2_/85% humidity.

### Characterizing iPSC lines

Pluripotency for all iPSC lines was confirmed by immunocytochemistry using antibodies (Ab) against Tra-1-60, Tra-1-81, SSEA3 and SSEA4, which are expressed in pluripotent stem cells. In addition, the capacity to differentiate into all 3 germ layers was established by in vitro assays, as previously described [24, 35]. The markers desmin (mesoderm), α-fetoprotein (endoderm), and βIII-tubulin (ectoderm) were used [22, 37, 38]. A list of the antibodies used to evaluate the iPSCs can be found in Additional file 2. Karyotyping was carried out by Cell Line Genetics (Madison WI). Each iPSC line used in this study had a normal karyotype, but each patient harbors the large, ~3 Mb deletion on 22q11.2, which was identified by FISH using a TUPLE probe or microarray [34].

### Neuronal differentiation

Neurons were generated from iPSC-derived neural progenitor cells (NPCs) as described by Marchetto et al. with slight modifications [26, 35]. A detailed description of the protocol can be found in Additional file 2. Essentially, the protocol leads to a mixed population of glutamatergic and GABAergic neurons (see Additional file 2), while the ratio of the two neuronal types and subtypes likely vary among samples due to the complexity of differentiation (see below). Neuronal samples were harvested on day 14 following differentiation from NPCs, and RNA was extracted and sent for sequencing. As such, the neurons used here were largely at their early differentiation stages and had not reached the stage that action potentials could be detected.

### Quantitative real-time PCR (qPCR)

qPCR was carried out on reverse transcribed cDNA from the same RNA samples used for the RNA-seq by the 2^-ΔΔCt^ method as previously described [39]. A detailed description and the primers used for this analysis can be found in Additional file 2.

### Proliferation assay

Cell proliferation was assayed using the Vybrant MTT cell proliferation assay kit (Invitrogen) according to the protocol manual. Briefly, and equal number of NPCs (10,000 cells in triplicate) were seeded on 96 well plates coated with poly-L-ornithine hydrobromide and laminin (day 0). Cell counts were determined daily for 7 days. At the time of the assay, 100ul of medium was removed from the well and replaced with an equal volume of fresh medium without FGF2, along with 10ul of the 12 mM MTT (3-[4,5-dimethylthiazol-2-yl]-2,5-diphenyltetrazolium bromide) stock solution. The cells were incubated at 37 °C for 4 h. 85 ul of medium was removed and 50 ul of DMSO was added, followed by a 10-min incubation at 37 °C. The samples were mixed well, transferred to a microplate, and the absorbance at 540 nm was determined. A total of 8 NPC lines were analyzed; 4 controls and 4 with 22q11.2 del. The fold change in cell number was compared to the day 1 pooled control samples, which was normalized to 1.0. Statistical significance of pooled controls and pooled 22q11.2 del samples was determined at each day of growth using a Student’s t-test (2-tailed).

### RNA-seq data acquisition

Paired-end RNA-seq was carried out on an Illumina HiSeq 2000. We obtained 101-bp mate-paired reads from cDNA fragments with an average size of 250-bp (standard deviation for the distribution of inner distances between mate pairs is approximately 100 bp). RNA-seq reads were aligned to the human genome (GRCh37/hg19) using the software TopHat (version 2.0.8) [40]. We counted the number of fragments mapped to each gene annotated in the GENCODE database (version 18), which included multiple categories of annotated transcripts [41], and quantified transcript abundance as FPKM (fragments per kilobase of exon per million fragments mapped).

### Sample clustering and “Batch” correction

RNA-seq samples were clustered based on all expressed transcripts (average FPKM > 1, *n* = 17,669, Additional file 3: Figure S1A) or a list of selective neural stem cell and differentiating neuronal markers obtained from R&D Systems (http://www.rndsystems.com/molecule_group.aspx?g=824&r=7) (*n* =55; Additional file 3: Figure S1B). We performed UPGMA (unweighted pair group method with arithmetic mean) clustering of samples from transcriptomic profile similarities based on the Pearson correlation coefficients. The analysis showed that our samples could be separated into two clusters; the cluster membership did not change whether all transcripts or only the neural marker genes were used for clustering. The first cluster (left on the heatmaps in Additional file 3: Figure S1B) exhibited higher expression of neuronal markers (e.g., *TUBB3* and *MAP2*), while the second (right on the heatmaps in Additional file 3: Figure S1B) showed greater expression for neural stem cell and neural progenitor markers (e.g., *VIM*, *SMAD*2 and *NOTCH2*). These suggest that variation in the degrees of neuron differentiation and maturation existed in our samples and needs to be accounted for in the differential expression analysis. Note that all of our samples showed a characteristic expression pattern for neuronal samples (higher expression of neuronal markers and lower expression of NPC markers) when compared with the NPC samples derived from a subset of our control iPSCs previously [42] (data not shown). Therefore, we considered the two clusters as two “batches” and applied ComBat [43] (a batch-correction tool adjusting for differences in the means across the batches and the variances, which would not be considered in a standard two-factor analysis) to correct the raw gene expression values and used the “batch”-corrected values for all subsequent analysis. Note that heterogeneity in neural induction is a rather common issue that has been discussed previously not only for our protocol [26], but also for others [44, 45], and the usage of multiple iPSC clones/lines from the same individuals has been suggested [46–48].

### Characterization of neuronal fate and maturity

To characterize the neuronal fate and maturity of our differential neurons, we compared gene expression profiles of our samples with two independent datasets. The first one is based on single cell RNA-seq analysis of human adult cortical samples in which all major cell types (astrocytes, endothelial, microglia, neurons, oligodendrocytes and oligodendrocyte progenitors) of the adult brain were identified [49]. Using the top 5,000 most variable genes in this dataset (which were enriched for signature genes in different cell types) [50], we performed non-metric multidimensional scaling after normalizing expression data across samples and batch correction. The plot of the transformed data in the first two dimensions (Additional file 4: Figure S2A) indicates that our neuronal samples were most similar to populations of adult neurons, with no separation of the 22q11.2 deletion samples from controls. The second one is a temporal gene expression data set encompassing cerebral cortical development from human embryonic stem cells [51], which classified their RNA-seq samples to five developmental stages: “Pluripotency” (PP: Day 0), “Neural Differentiation” (ND: Day 7), “Cortical Specification” (CS: Day 12), “Deep Layer neuron generation” (DL: Day 26), and “Upper Layer neuron generation” (UL: Day 63). Principal component analysis (PCA) indicates that our samples were mapped to a stage between Day 12 (the start of the CS stage) and Day 19 (Additional file 4: Figure S2B, PC1), suggesting that our samples likely had passed the peak of neuron differentiation and were undergoing specification of neuronal subtypes. Again, in this analysis our control and SZ samples were grouped together and mapped to the same differential stages. Finally, we looked into a number of markers for glutamatergic and GABAergic neurons, and astrocytes, which were expressed at least in one of our samples (Additional file 4: Figure S2C), and found that glutamatergic and GABAergic markers had relatively high expression levels, while markers for astrocytes and other subtypes were either not expressed or expressed at relatively low levels.

### Identifying differentially expressed genes (DEGs)

Although we have biological replicates for a few iPSC lines, we found that intra-individual variations were as large as inter-individual variations. The correlation of coefficients (CV) for two controls with duplications were 0.25 and 0.28 and for two SZ with duplications were 0.17 and 0.34. These numbers were very similar to the CVs observed for inter-individuals, 0.26 for controls and 0.28 for SZ samples. As such, we analyzed all samples together without specifically weighting or nested analysis of the samples derived from the same individuals.

We used DESeq2 [52] to determine differential expression from the corrected RNA-seq read count values, analyzing only transcripts with an average FPKM ≥ 1 across all samples. Considering that neuropsychiatric disorders, including SZ, are highly heterogeneous, we determined statistically significant differences in gene expression between SZ samples and controls at a nominal *p* value (*p* < 0.05), an approach similarly taken in many previous SZ transcriptomic studies [25, 53–58], but we also applied a multiple comparison correction to the *p*-values to compute for false discovery rate (FDR) [59].

### Identifying differentially expressed gene modules

We used Weighted Gene Coexpression Network Analysis (WGCNA) [60] to identify co-expressed gene modules from all of our RNA-seq data. From the WGCNA modules, we identified differentially expressed modules according to the recommendation by the developers of the software. To do that, we utilized the module preservation statistic (*Z-summary*), which takes into account both the overlap in module membership and the density and connectivity patterns of modules, to assess the module preservation between the control and SZ samples. Technically, *Z-summary* < 2 implies no evidence for module preservation, 2 < *Z-summary* < 10 implies weak to moderate evidence, and *Z-summary* > 10 implies strong module preservation. In order to obtain networks of high connectivity and minimize the adverse effect of a moderate sample size, we constructed networks as follows: first, we constructed a network from the combined case and control data and identified modules within it; then, we only tested modules with preservation *Z-summary* > 10 between control and SZ samples for differential expression.

### Function enrichment analysis

We identified enriched pathways in the REACTOME databases with Toppgene [61] and enriched Gene Ontology (GO) terms with DAVID [62, 63].

### Mapping differentially expressed genes to BrainSpan transcriptomes

To evaluate gene coexpression across brain regions at different developmental periods for the DEGs from our 22q11.2 del samples, we reanalyzed the RNA-seq gene expression data from the Atlas of the Developing Human Brain (BrainSpan) (http://www.brainspan.org). We performed correlation analysis to uncover genes co-expressed with our DEGs. As described in previous studies [64], tissue samples from the BrainSpan were grouped into four neuroanatomical regions—frontal cortex (FC), temporal and parietal regions (TP), sensory-motor regions (SM), and subcortical regions (SC), and four developmental stages—embryonic stage (8–12 post-conception weeks), fetal (13–26 post-conception weeks), early infancy to late childhood (4 months to 11 years), and adolescence to adulthood (13–23 years) (Table 1). Next, for each of the DEGs, we calculated Pearson correlation coefficients (R) between its expression and that of all other DEGs across tissues within each brain region and at each developmental stage. 46 DEGs were excluded since their expression values were zero in all tissues. Gene pairs with |R| ≥ 0.9 were defined as significantly co-expressed and thus “connected” in subsequent co-expression construction. Network connectedness was measured by the number of connections, i.e., edges [65]. The value of 0.9 was used because the resulting connections exhibited an expected power law feature after testing the choice between 0 and 1 [66]. To generate null distribution of connectedness, we performed 10,000 iterations of the same analysis on the same number of genes that were randomly picked to have their FPKM distribution similar to that of DEGs in each region and stage. More specifically, we ranked genes within each data group according to their expression levels, and then randomly selected one gene whose rank was within 5 of each DEG, to ensure that we avoid potential bias due to differences in expression as genes of lower expression are less likely to form connections with others. Afterwards, we tested if connectedness of DEGs was significantly deviated (one-tailed, larger) from the null hypothesis by simulation. A multiple comparison correction was used to correct *p*-values [59].

**Table 1:** Number of tissues by brain region and developmental stage used in network analyses [64]

			Number of specimens			
Brain Region		Tissue	8–12 pcw	13–26 pcw	4mon–11Y	13–23 Y
FC	DFC	Dorsolateral prefrontal cortex	18	38	37	19
	MFC	Anterior (rostral) cingulate (medial prefrontal) cortex				
	OFC	Orbital frontal cortex				
	VFC	Ventrolateral prefrontal cortex				
SC	STR	Striatum	13	34	32	18
	MD	Mediodorsal nucleus of thalamus				
	AMY	Amygdaloid complex				
	HIP	Hippocampus				
SM	A1C	Primary auditory cortex (core)	14	36	32	19
	M1C	Primary motor cortex (area M1, area 4)				
	S1C	Primary somatosensory cortex (areas S1,3,1,2)				
	V1C	Primary visual cortex (striate cortex, area V1/17)				
TP	ITC	Inferolateral temporal cortex (area TEv, area 20)	10	28	32	18
	STC	Posterior (caudal) superior temporal cortex (area TAc)				
	IPC	Posteroinferior (ventral) parietal cortex				

*FC* frontal cortex, *SC* sub-cortical, *SM* sensory-motor, *TP* temporal-parietal

8–12 pcw: embryonic (stage1); 13–26 pcw: fetal (stage2); 4mon–11Y: infancy to childhood (stage3); 13–23Y: adolescence to adulthood (stage4)

Likewise, we determined the Pearson correlation coefficients between each of the DEGs and the remaining genes to assess the potential major biological implications of DEG networks. We collected the top 10% of most connecting DEGs (*n* = 73) (i.e. the subset of DEGs showing connections to the largest number of other DEGs) and the top 10% of non-differentially expressed genes (*n* = 300) that had the most connections with DEGs. The function relationship and enrichment among these 373 genes were analyzed by the software ClueGO [67], with the resultant networks visualized by the Cytoscape [68].

## Results

### Differential gene expression analysis

**Fig. 1:**
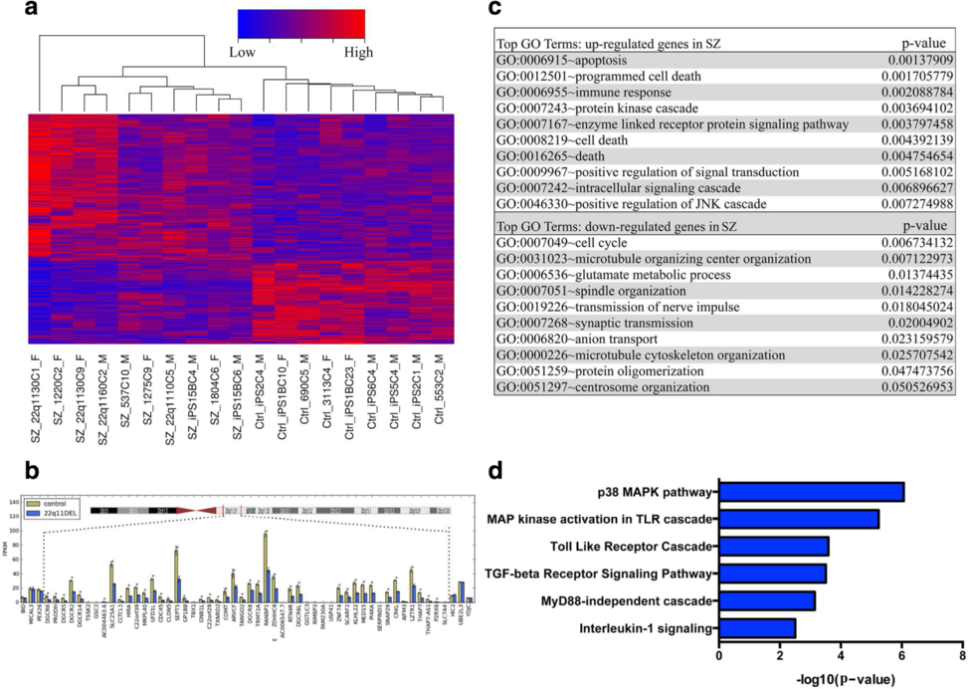
Differentially expressed genes in 22q11.2 SZ neurons and their enriched functions. **a** Heat map showing relative expression of 782 genes that exhibited significant change between control and SZ at *p*-value < 0.05 (503 increased in SZ; 279 decreased). **b** Bar plot presenting batch-corrected expression values of 22q11.2 genes in control and SZ samples. Three genes flanking the deleted region at either side were also included. Asterisks (*) indicate significant differential expression at the genome-wide level (FDR < 0.05). **c** Enriched GO terms of the DEGs as determined by the software David. **d** Enriched pathways for the DEGs as determined by the software Toppgene

We generated eight and seven iPSC lines from 22q11.2 patients and healthy individuals, respectively, and obtained RNA-seq data from a total of 19 differentiating neuron samples (seven controls with two duplicates for a total of nine samples; eight 22q11.2 DS SZ and SAD patients with two duplicates for a total of ten samples). The number of reads obtained from the RNA-seq runs for each of the 19 samples and the fraction that could be aligned to the human genome were comparable (Additional file 5). A total of 14,549 transcripts were expressed in our samples, including 12,981 protein-coding and 512 lincRNAs (long intergenic non-coding RNAs). Clustering analysis of the samples based on their raw FPKMs yielded two groups, which likely reflects heterogeneity in neural differentiation (see Methods for details). We then applied a batch correction method to account for the expression variation and used the corrected expression values in the software DESeq2 [52] to determine differential gene expression. The entire gene list with corrected expression values can be found on Additional file 6. After filtering out low expressed genes (mean FPKM < 1 across 19 samples), we identified 782 differentially expressed genes: 503 increased in the SZ samples and 279 decreased, at nominal *p* < 0.05 (Additional files 7 and 8, respectively) (Fig. 1a). Because of the relatively moderate sample size and experimental variation, only a small number of differentially expressed genes were statistically significant after correcting for multiple testing (42 genes by FDR < 0.05). Nevertheless, based on the FPKM values and expression changes for genes in the 22q11.2 deleted region, we considered *p* < 0.05 a reasonable threshold for calling differential expression. As seen in Fig. 1b, 36 of the 47 protein-coding genes in the 22q11.2 region, including most of the candidate genes implicated in the psychiatric manifestations or endophenotypes associated with 22q11.2DS (*DGCR8*, *DGCR2, RANBP1, RTN4R*, and *COMT*, for example) showed a significant decrease (~2-fold reduction, FDR < 0.05) in the patient samples compared with controls. One exception is *CLDN5*, which also showed larger than 2-fold difference in expression between conditions, but failed to reach statistical significance because of large intragroup variability and relatively lower expression. Another exception is *TBX1.* Its expression in the neuronal differentiation method we used is even lower (mean FPKM ~0.6 in control and 22q11.2 del neurons), so accurate quantitation is difficult. The other differentially expressing genes passing FDR <0.05 but not in the 22q11.2 region are *DDX11, PDK3, PCIF1, FAM103A1, TMSB4X* and *HLA-A*.

Note that the three genes flanking either side of the deleted region were not differentially expressed (Fig. 1b). In fact, of the 311 genes on chromosome 22 that were outside of 22q11.2 and had a mean >1 FPKM in either the control or patient groups, 169 were expressed at a higher level in the patient samples; 142 were lower. Of these, only 14 genes showed a significant difference between patients and controls at *p* < 0.05 (7 were decreased in patients; 7 increased). The difference between significantly down-regulated genes on 22q11.2 compared with the number of significantly differentially expressed genes on the rest of chromosome 22 was highly significant (χ2 test *p*-value < 2.2e-16).

These data establish that haploinsufficency at 22q11.2 is recapitulated at the RNA expression level in our differentiating neurons and supports the validity of the RNA-seq data. We should mention that haploinsufficency at 22q11.2 could also be detected without applying the batch correction (Additional file 9). Our finding is consistent with previously studies that have also shown a 2-fold reduction of the 22q11.2 genes in both 22q11.2 DS patients and mouse models [69–71]. It is also consistent with our recently published findings that all of the miRNAs that are expressed in neurons that map to the 22q11.2 deleted region are significantly down-regulated in the patient samples *vs* controls [34].

We should mention that there were no significant differences in the expression of the glutamate vesicular transporters (*SLC17A6* and *SLC17A7*), or the GABA transporter (*SLC6A1)* in patient vs control neurons (Additional file 6), suggesting that the overall population of GABA and glutamate neurons were similar. Also note that the GABA transporters *SLC32A1, SLC6A13,* and *SLC6A1* are not expressed in these early differentiating neurons.

The differentially expressed genes (DEGs) were loaded into the software DAVID and ToppGene to identify enriched gene pathways and networks. The top enriched GO terms for the genes that showed an increase in expression in the SZ neurons were apoptosis/programmed cell death and immune response, while the top GO terms for the genes that decreased in expression in the SZ neurons were cell cycle, microtubule organizing center organization and glutamate metabolic process (Fig. 1c). From pathway analysis by ToppGene, the top canonical pathways for all DEGs are involved in the MAPK signaling cascade (Fig. 1d), such as the p38 MAPK and Toll-like receptor pathways. This finding is consistent with a previous genome-wide transcriptome analysis of peripheral blood mononuclear cells (PBMC's) from 22q11DS SZ patients [69], in which ERK/MAPK signaling was also identified as one of the top canonical pathways disrupted in patients. In our data, significantly elevated expression was observed for several MAPK encoding genes (*MAP3K2, MAP3K7* and *MAP3K6*) and related factors (*JUN, PRKCD, HOMER3* and *MYH9*). In addition, expression levels of upstream regulators for the PI3K/AKT signaling pathway, *PIK3C2A* and *PPM1F,* were increased as well. These results suggest that disruption of the MAPK signaling cascade in 22q11.2 SZ neurons might result in prolonged cycles of cell division and cell proliferation, and enhanced cell death through apoptosis during neuronal differentiation.

In the above expression analysis, we have treated duplicated samples from the same subjects as independent samples. We also carried out an analysis by first averaging the expression values of the duplicated samples and then running differential expression analysis. This resulted in 513 DEGs (451 overlapped with the above list of DEGs), which showed similar function and pathway enrichments: MAPK signaling, apoptosis and cell cycle.

Thirteen up-regulated and 13 down-regulated genes outside of the 22q11.2 region were among the list of 883 suspected SZ candidate genes obtained from Schizophrenia Forum (http://www.schizophreniaforum.org). Interestingly, out of the 13 up-regulated SZ candidates, 8 have been implicated in apoptosis (*CD4, CFLAR, HOMER3, MYH9, NDRG1, PIK3C2A, PPM1F* and *UHMK1*).

### Validation of RNA-seq data

**Fig. 2:**
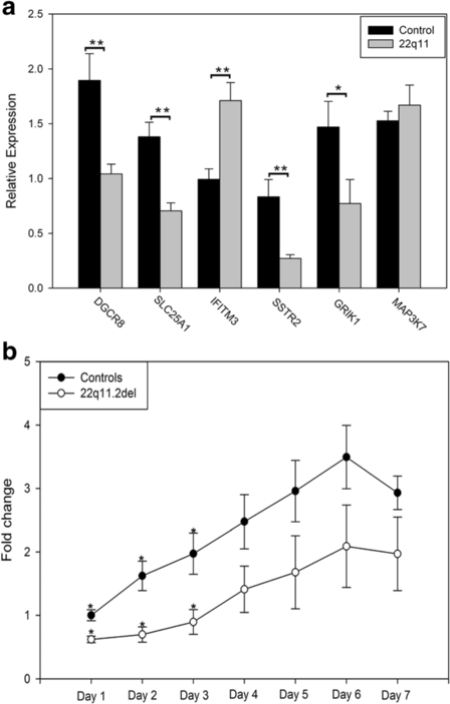
qPCR validation and NPC proliferation assay. **a** qPCR analysis of six DEGs using neuronal RNAs from 4–5 controls and 4–5 patients. Relative expression values were determined and pooled by phenotype. Error bars show standard error of the mean; Two-tailed t-test *p*-value <0.01 (**) and <0.05 (*) are indicated. **b** Proliferation rates of NPCs derived from control (*n* = 4) and 22q.11.2 (*n* = 4) iPSCs. An equal number of cells were plated for each sample (10,000) on day 0. Fold changes were calculated by normalizing the day 1 controls to 1.00, and the control and patient samples were pooled. Statistical significance was determined using a two-tailed Student’s t-test. The asterisk denotes statistically significant difference in proliferation at *p* < 0.05

We used qRT-PCR to validate the RNA-seq differential gene expression profile for 6 genes (Fig. 2a). Two of the selected genes map to the 22q11.2 deleted region – *DGCR8* and *SLC25A1*. Both showed a statistically significant, ~2-fold decrease in expression, supporting the RNA-seq findings for these genes. The others were analyzed because of their relevance to SZ and our findings in pathway analysis. These included *IFITM3, SSTR2, GRIK1,* and *MAP3K7. IFITM3* codes for interferon-induced transmembrane protein 3, which plays a role in interferon-signaling and the innate defense against influenza and other viruses [72–74]. This is interesting from a SZ pathogenesis perspective, considering the clinical and epidemiological evidence pointing towards prenatal influenza as a risk factor in SZ [75, 76]. *SSTR2*, which codes for somatostatin receptor 2, is important because reduced expression in the cortex, and a reduction in somatostatin positive GABAergic hippocampal neurons has been detected in SZ brains [77–79]. Finally, *GRIK1*, which codes for a kainite glutamate ionotropic receptor, is expressed at lower levels in SZ brains [80]. As seen in Fig. 2a, the significant changes in expression found by RNA-seq were confirmed by qPCR.

We also tried to confirm the increased expression of *MAP3K7* mRNA detected in the SZ neurons by RNA-seq. Although a similar increase in expression was found in the SZ neurons – 16.3% for the RNA-seq and 13.3% for qPCR - the latter was not statistically significant. This was not especially surprising since the relatively small increase in *MAP3K7* expression found in the RNA-seq analysis challenges the sensitivity of qPCR. It should be noted, however, that the enrichment of genes involved in MAP kinase signaling detected in our pathway analysis was likely due to the combination of small differences in the expression of many genes, rather than a large difference in expression in a small number of genes.

In order to determine if the GO assessment showing a decrease in the expression of genes associated with the mitotic cell cycle was accompanied by a decrease in cell growth, we assessed the proliferation rate of NPCs. Cell growth was assayed in 8 NPC lines (4 controls and 4 with 22q11.2 del). The fold change data were pooled, and control vs 22q11.2 del growth rates were analyzed as described in the methods section. As seen in Fig. 2b, there was an overall decrease in proliferation for the 22q11.2 samples at each time point. However, because of line to line variability, statistically significant differences were only seen on days 1–3 (two-tailed Student’s t-test, *p* = 0.01 on days 1,2; 0.03 on day 3; 0.11, 0.14 0.14 and 0.18 on days 4–7, respectively). Nevertheless, the proliferation data support the GO enrichment findings from our RNA-seq data.

### Weighted Gene Coexpression Network Analysis (WGCNA) for differentially expressed gene modules

**Fig. 3:**
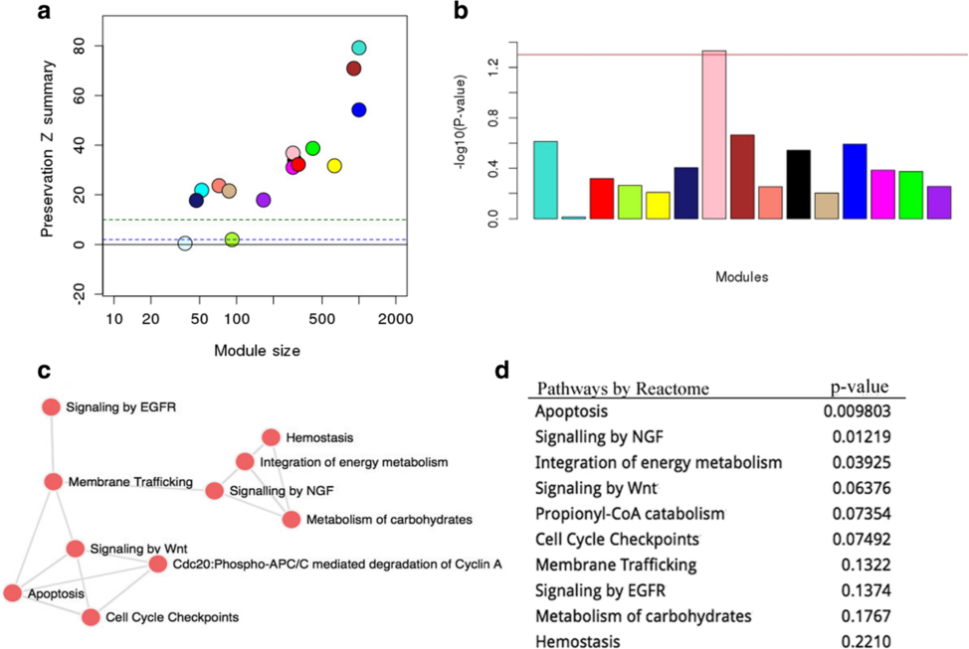
WGCNA modules and function enrichment. **a** Module preservation Z-summary statistics of 15 modules identified by WGCNA. **b** Barplot showing the –log10 (*p*-value) (*y-axis*) of expression differences between SZ samples and controls for 13 modules whose module structures were well preserved between SZ and control samples (Z > 10, A). **c** Network of top pathways in the differentially expressed pink module. Each node represents a term and an edge between two nodes indicates that the two terms share genes. **d** The top pathways in the pink module as determined by the software Enrichr

The above analysis was focused on the characterization of individual genes whose expression was affected by 22q11.2 deletion. It is equally, if not more important, to uncover gene networks that may be disrupted in the SZ neurons, as many genes function together whereas they may not all show significant expression change. We thus performed WGCNA analysis and detected 15 modules, 13 of which showed no significant differences in module structure between the two conditions (i.e, preserved modules) (Fig. 3a). The two modules below preservation thresholds (light-cyan and green-yellow) had very small numbers of genes. The light-cyan module contained 38 genes in total, 36 of which were from 22q11.2 deleted region; thus its lack of preservation is expected. The green-yellow module was comprised of 92 genes, most of which had an extreme outlier of expression values from one particular control sample (Ctrl_iPS2C). The outlier was the cause of low preservation score. Therefore, the WGCNA results suggest that there is no global gene regulation re-wiring in the SZ neurons. This is in accordance with three previous gene network studies carried out on patients with psychosis [81, 82], which also did not observe a significant perturbation of gene modules in patient samples.

Next, we searched for WGCNA modules that showed significantly differential gene expression between SZ neurons and controls. Only one module (pink module with 289 genes) was found (*p*-value < 0.05) (Fig. 3b). Note that 99 of the 289 genes were in our list of DEGs. *PLAU* (plasminogen activator, urokinase) and *PPP2R1B* were the two top hub genes in the pink module (Table 2); both showed increased expression in SZ neurons. *PLAU* and its receptor (*PLAUR/uPAR*) are mainly involved in invasion and cell proliferation, and their increased expression is correlated with a wide range of human diseases, including autism, Alzheimer’s, AIDS dementia, cerebral malaria and brain tumors [83]. Their anti-apoptotic effects occur via the caspase-mediated apoptotic pathway and the PI3K/AKT pathway [84]. Markedly elevated levels of uPA/uPAR expression has been reported in chronic neurodegeneration, AIDS dementia complex and other neurological disorders, suggesting that the *uPA/uPAR* system may contribute to neuronal damage [85, 86]. *PPP2R1B* encodes a regulatory subunit of protein phosphatase 2 (*PP2A*), which is involved in the negative control of cell growth and division *via* the PI3K/AKT pathway [87]. *PPP2R1B* itself has also been implicated in deregulation of cell cycle and apoptosis in B-cell chronic lymphocytic leukemia [88].

**Table 2:** Top hub genes of the pink WGCNA module

Gene	Pink Module Membership^a^	log2_fold_change(SZ/Ctrl)	DE *p*-value
*PLAU*	1.61E-10	1.386071927	0.0010647
*PPP2R1B*	4.03E-10	0.877010644	0.0003536
*FXYD5*	8.24E-10	1.051702956	0.0418776
*MYSM1*	1.44E-09	0.321646132	0.0026121
*TPCN1*	4.75E-09	0.324961243	0.0391725
*DOCK5*	5.82E-09	1.151201505	0.0017714
*SAR1A*	6.27E-09	0.218170473	0.0825168
*DNAJC21*	6.28E-09	0.260891882	0.0765025
*PRDM5*	7.87E-09	0.514186001	0.0412623
*ENTPD4*	1.05E-08	0.308259161	0.002583

^a^Module membership of each gene is measured by testing significance of correlation between its gene expression and the module eigengene of a given module

The potential importance of the pink module in apoptosis was supported by functional enrichment analysis. Using the RECTOME database with Enrichr [89], we found that the pink module was significantly enriched with genes that regulate apoptosis (Fig. 3c/d), consistent with the functions of the two hub genes described above. These results suggest that 22q11.2 deletion may disrupt normal apoptotic activity, by affecting one of the MAPK signaling pathways, the PI3K/AKT pathway.

### Potential effects of 22q11.2 deletion via inter-chromosomal interaction

**Fig. 4:**
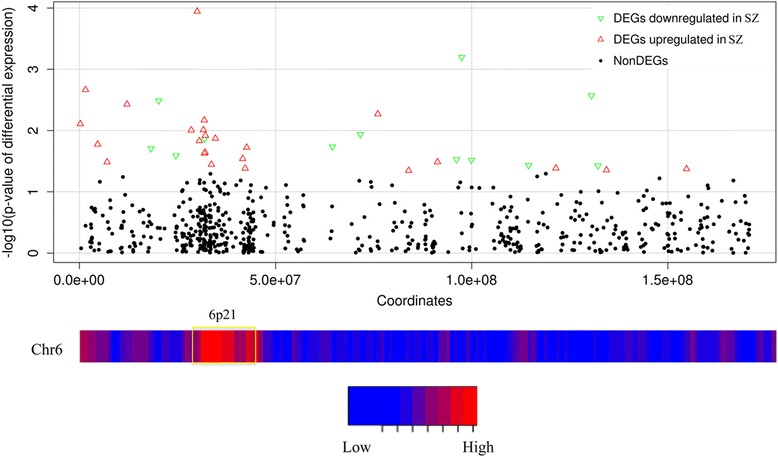
Enrichment of DEGs in a region of chromosome 6 predicted to interact with 22q11.2. Scatterplot presenting the –log10 (*p*-values) (*y-axis*) of changes in gene expression for each gene along chromosome 6. The heat map below shows the reported Hi-C correlation between each section of chromosome 6 and the 22q11.2 region. Note that the 6p21 region, which is highlighted by a *yellow box* in the heat map, was enriched for differentially expressed genes, and it displayed the strongest physical interaction with 22q11.2

Some DEGs are downstream of signaling pathways perturbed in the SZ neurons, but the dysregulation of others may be due to disrupted spatial chromosomal interactions, which occur in the nucleus under normal physiological conditions, juxtaposing distal genes for efficient co-regulation [90–94]. By analyzing Hi-C data generated to assess chromatin folding and packaging in the nuclei of human lymphoblastoid cell lines [95], we identified three genomic regions with the strongest physical interaction with 22q11.2: 4p16, 8q24 and 6p21 (mean Pearson correlation coefficient > 0.4). Among the three, only 6p21 was statistically enriched for differentially expressed genes (χ2 test *p*-value = 0.01) (Fig. 4). Interestingly, this region has recently been linked with 16p11.2 deletion syndrome, a genetic cause for ASD [96]. 6p21 contains a number of genes involved in immune responses, including the human leukocyte antigen (HLA) gene cluster. As mentioned earlier, one of the *HLA* genes residing in this region, *HLA-A*, was among the small group of DEGs that remained statistically significant at a genome-wide scale. It should also be pointed out that the most robust GWAS (genome wide association studies) signals in SZ map to the *HLA* locus [97–99]. Although the physical interaction between 22q11.2 and 6p21 needs to be confirmed and further characterized, our findings suggest that 22q11.2 deletion may affect *HLA* gene expression through direct long-range contact in *trans.* In this regard, we should mention that abnormal expression of immune response genes, especially *HLA* genes in 6p21, has been often suggested to be a common factor underlying neurodevelopmental disorders [96, 100]. It should be noted, however, that HLA proteins have been suggested to have non-immune effects on synaptogenesis [101–105].

### Spatiotemporal expression of the DEGs during brain development and the co-expressed networks

Next, we set out to address how our findings from in vitro neuronal cultures could be related to brain development. In particular, we asked what genes and pathways that are expressed during normal brain development could be impacted if the same set of genes were dysregulated in the brains of individuals with 22q11.2DS.

**Fig. 5:**
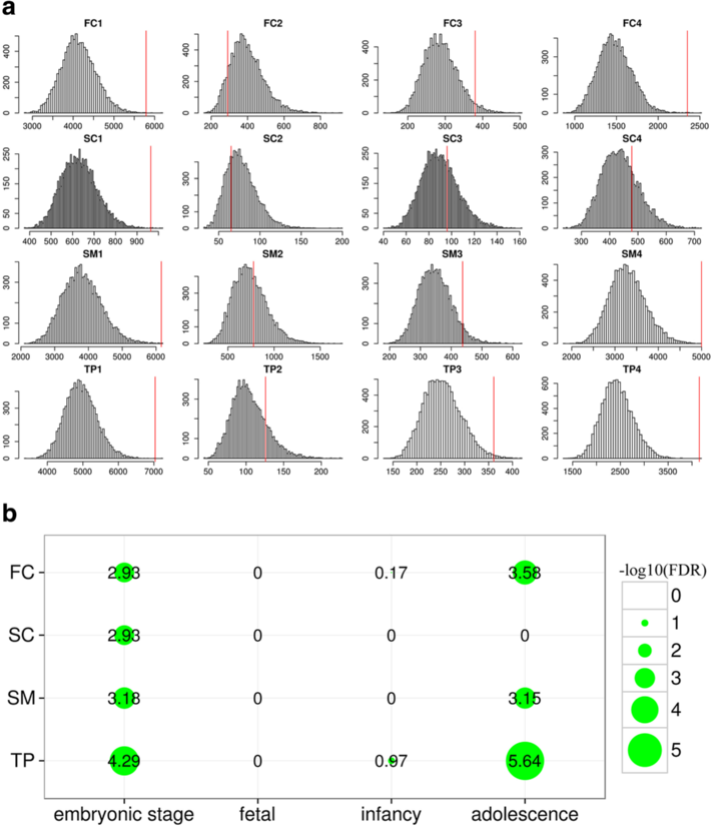
Highest co-expression of DEGs in specific brain regions. **a** Interconnectedness of transcriptional coexpression networks at various developmental stages and in different brain regions based on DEGs or randomly selected genes were evaluated using RNA-seq data from the BrainSpan Atlas. A pair of genes was defined as coexpressed if their expression correlation coefficient |R| was ≥ 0.9, across all tissues from a given brain region and a given developmental stage (Table 1). Dotted lines indicate numbers of connections (i.e., edges) in networks actually observed for DEGs, while the histograms represent distributions of the numbers of edges in 10,000 simulated networks derived from randomly picked genes. **b** Multiple testing adjusted –log10(*P*-value) for the significance of difference between the numbers of observed and simulated network edges

First of all, we reasoned that the 782 DEGs or a subset of them would form co-expressed network(s), and that perturbation of the network(s) could contribute to functional disruptions in SZ patients with 22q11.2 deletion, as co-expressed genes are often functionally associated. To address this, we obtained gene expression data from the Brainspan project and separated the Brainspan samples (*n* = 398) into 16 groups according to brain regions and developmental stages (see Methods for details) (Table 1). Within each group, we computed the correlation coefficient of expression between every pair of our DEGs and connected a pair of genes if the coefficient was > 0.9 (or < −0.9), resulting in a co-expression network. In analyzing the networks, we found, remarkably, that the DEGs from our study showed high levels of connections in brain regions of two developmental stages, the embryonic and the adolescence stages (Fig. 5a). Moreover, the numbers of edges in the networks for all four embryonic brain regions and three of the four adolescence regions (except subcortex) were significantly greater than those of the networks derived from randomly chosen genes (Fig. 5b). These results indicate that a subset of the 22q11.2 DEGs are significantly co-expressed and likely functionally connected to two key stages of brain development.

There are at least three hypotheses to explain how 22q11.2 haploinsufficency could cause the cognitive and psychiatric phenotypes of SZ: i. unmasking recessive alleles in the deleted region, ii. a major effect of a single dosage-sensitive gene, and iii. under-expression of several dosage-sensitive genes [106]. Existing data from animal models and systematic genetic association studies seem to support the under-expression model [8, 107]. The co-expression networks for our DEGs also support this hypothesis, as a small number of DEGs accounted for the majority of the connections in the networks (Additional file 10) and moreover only a few of the 22q11.2 genes were among the highly connected genes (Table 3). In addition, the most connected genes varied from brain regions and developmental stages. In general, *CDC45* had the largest number of connections in the embryonic stage while *PRODH* was among the five most connected genes in the adolescence brain regions (Table 3). Note that the ranks of DEGs according to their numbers of connections in the co-expression networks exhibited better correlations in brain regions of the same developmental stage (Table 4). The data indicate that the co-expression networks display greater differences between developmental periods than brain regions, further suggesting that different subsets of the DEGs may play important roles at different periods of brain development.

**Table 3:** DEGs or 22q11.2 genes with the most co-expressed genes in different brain regions*

Brain Region	Embryonic		Adolescence	
	DEGs	22q11.2	DEGs	22q11.2
FC	*KIAA1467*	*CDC45*	*HERC3*	*CRKL*
	*MAPKAPK3*	*COMT*	*DDX3X*	*PRODH*
	*CHD1L*	*TXNRD2*	*MTMR7*	*DGCR6*
	*SEPT2*	*SLC25A1*	*ELL2*	*LZTR1*
	*RFXANK*	*THAP7-AS1*	*LRRTM3*	*C22orf29*
SC	*SCAF11*	*CDC45*	*PJA2*	*C22orf39*
	*KHNYN*	*SLC25A1*	*ZMYM2*	*MED15*
	*MAPKAPK3*	*CLDN5*	*DPP8*	*PRODH*
	*TP53*	*COMT*	*PDCD6IP*	*HIRA*
	*LAMC3*	*CRKL*	*B4GALT6*	*TANGO2*
SM	*PLIN3*	*CDC45*	*PDCD6IP*	*COMT*
	*FGFR2*	*CRKL*	*HDAC7*	*DGCR6*
	*RREB1*	*THAP7-AS1*	*UBE3C*	*CRKL*
	*MAPKAPK3*	*COMT*	*LRFN5*	*PRODH*
	*MCAM*	*SEPT5*	*GLRB*	*SLC25A1*
TP	*FGFR2*	*SLC25A1*	*MEF2A*	*COMT*
	*MAP7D3*	*CDC45*	*NRXN1*	*DGCR6*
	*PLIN3*	*RANBP1*	*FBXO45*	*CRKL*
	*MAPKAPK3*	*SCARF2*	*PDCD6IP*	*PRODH*
	*TP53*	*SNAP29*	*MTMR7*	*SLC25A1*
Total	*MAPKAPK3*	*CDC45*	*PDCD6IP*	*PRODH*
	*PLIN3*	*SLC25A1*	*MTMR7*	*DGCR6*
	*SCAF11*	*COMT*	*GLRB*	*COMT*

*In each stage and region, DEGs or 22q11 deleted genes were ranked according to their numbers of connection with other DEGs. The top three genes in the “Total” had the highest numbers of connections summed over all four regions in the embryonic or adolescence stages

**Table 4:** Correlation of ranking orders of DEGs according to their connectivity across brain region and stages

	SC1	SM1	TP1	FC4	SC4	SM4	TP4
FC1	0.4	0.69	0.57	0.06	−0.01	0.1	0.07
SC1		0.4	0.44	0.04	0.05	0.09	0.09
SM1			0.67	−0.05	−0.06	−0.05	−0.05
TP1				−0.08	−0.08	−0.06	−0.06
FC4					0.59	0.8	0.79
SC4						0.54	0.6
SM4							0.77

**Fig. 6:**
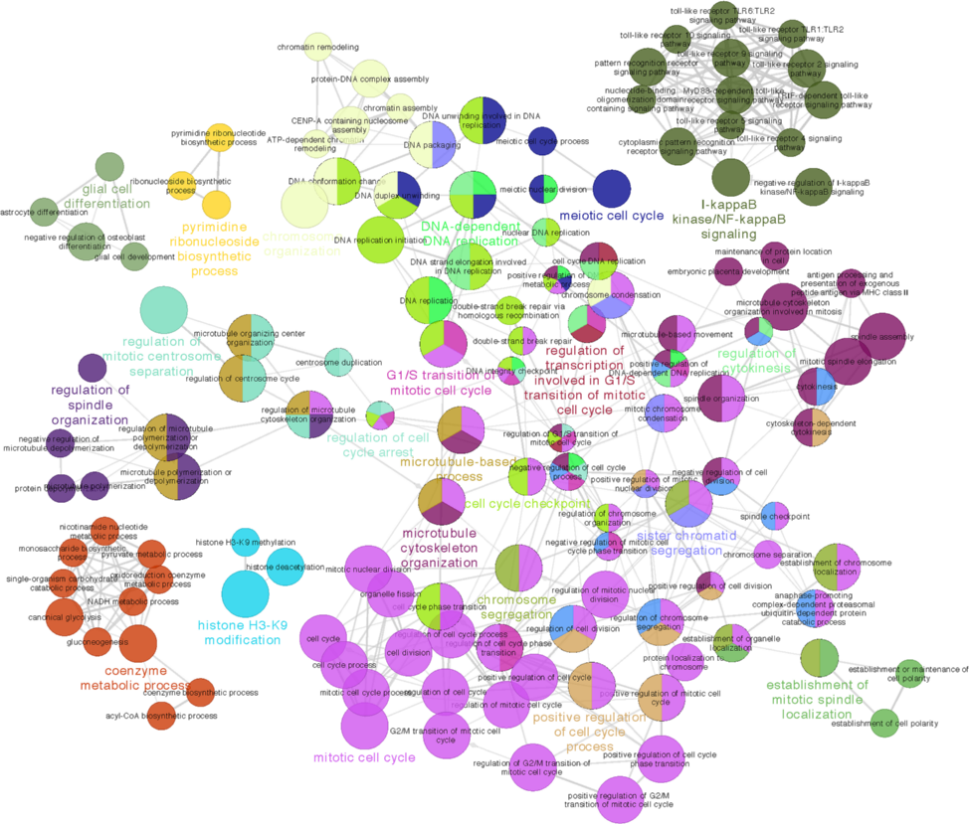
ClueGO network for top correlating DEGs and co-expressed non-DEGs in frontal cortex during the embryonic stage. The size of the nodes reflects the statistical significance of the terms. Only terms with *p*-values < 0.05 are shown. For grouping terms, the initial group size was set to 3 and the percentage for merging groups was set to 50%. A term can be included in more than one group. Different groups were colored differently. The group leading term (*in bold*) is the most significant term of the group

**Fig. 7:**
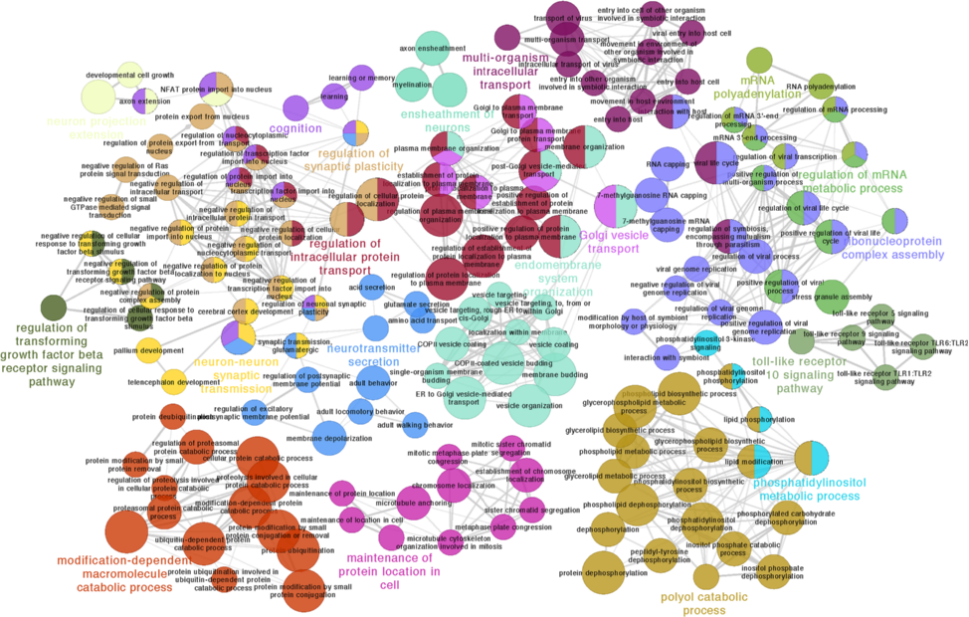
ClueGO network for top correlating DEGs and co-expressed non-DEGs in frontal cortex during the adolescence stage. See annotation in Fig. 6

To explore this further, we performed function enrichment analysis of the co-expression networks in the embryonic and adolescence brains. We expanded the networks to include non-differentially expressed genes that showed high co-expression correlations to the DEGs, because conceivably those genes were most likely to be affected by 22q11.2 deletion too. However, to reduce complexity we focused on functions of the top 10% of the most connected DEGs (*n* = 74) and the top 10% of non-DEGs with the greatest numbers of connections to the top DEGs (*n* = 300). In order to better illustrate the function relationships for the top 10% of connected genes, we utilized ClueGO [67] to define enriched GO terms and to visualize the non-redundant biological process GO terms (Figs. 6 and 7). This analysis uncovered several functional clusters of interest, which were different between the two developmental stages. For example, in the frontal cortex of the embryonic stage, the top 10% of connected genes were enriched with biological processes critical for cell cycle, cell differentiation, and cell growth in the embryonic stage (Fig. 6). Synaptic transmission and catabolic process, however, were enriched for the most connected genes in the frontal cortex of the adolescence stage (Fig. 7). Similar enriched functional clusters were observed in other brain regions of the two stages (Additional file 11). These results strongly suggest that different genes in the 22q11.2 region play important temporospatial roles in different periods of brain development and haploinsufficiency of 22q11.2 genes have distinct functional impacts in different brain regions during brain development. This is also manifested at least partially by the temporal expression profiles of the 22q11.2 genes in brain regions at different developmental stages (Additional file 12).

## Discussion

This is the first study of gene expression profiles in early differentiating neurons derived from patient-specific iPSCs that were generated from SZ and SAD patients with 22q11.2 deletion. Our transcriptomic analysis provides insight into the neuronal functional disruptions of 22q11.2 deletion. We show that 22q11.2 haploinsufficiency at the genetic level is recapitulated in RNA expression of in vitro neurons. At the molecular genetic level, GO and canonical pathway analyses of the differentially expressed genes implicate the potential disruptions of MAPK signaling, cell cycle and apoptosis in 22q11.2 SZ neurons. At the network level, we find that 22q11.2 genes and their co-expressed targets likely play two distinct roles during brain development, with a *CDC45* mediated cell cycle pathway involved in embryonic brain development and a *PRODH* modulated subnetwork contributing to adolescent brain functions. We also uncovered a potential interchromosomal interaction between 22q11.2 and 6p21, suggesting a molecular link between immune deficiency and additional disruption of synaptogenesis in 22q11.2 DS mediated by the non-immune function of HLA proteins on this process.

One of the most important and consistent findings that emerged from our transcriptomic profile was the enrichment of apoptotic genes in the DEGs. Aberrant apoptosis has been implicated in various neurodevelopmental and neurodegenerative disorders, including SZ [108–112]. Increased susceptibility to apoptosis in SZ patients may be responsible for synaptic/dendritic loss [113]. Reduced neuronal and glial viability, and volumetric and functional brain deficits observed in SZ are potentially associated with abnormal apoptosis [32]. Several studies have provided support to the hypothesis by showing increased brain *Bax/Bcl-2* ratios and decreased levels of *Bcl-2* and *GSK3* [114–116]. In addition, a comprehensive integrated pathway analysis of GWAS and gene expression data also pointed to aberrant apoptosis as a potential cause of SZ [110]. In our study, we found similar evidence. Apoptotic genes such as *Bak1* and *BBC3* had significantly higher expression in SZ neurons, while *GSK3A* showed lower levels of expression. Two critical apoptotic factors, *RBM5* and *RBM6,* also exhibited greater abundance in SZ neurons as well. Although statistically insignificant, *CASP3* and *CASP8* were also expressed at higher levels in the SZ samples. These findings, as well as differential expression of *TP53*, *TP53INP1*, and *NDRG1,* which participate in the P53 signaling pathway, and the WGCNA results, are all in agreement with the hypothesis of increased susceptibility to apoptosis in SZ brains.

Apoptosis is a complex cellular process involved in many steps of development and regulated by multiple pathways. Its activation is triggered mainly through two pathways: extrinsic (or death receptor) and intrinsic (or mitochondrial) pathways [113]. In addition, apoptosis is regulated by a number of intracellular signaling pathways, including MAPK signaling. In the CNS, MAPK signaling regulates the expression of transcription factors involved in learning, memory, cell proliferation, and apoptosis. This pathway responds to extracellular stimuli by phosphorylating c-Jun N-terminal kinase (JNK), extracellular signal-regulated kinase (ERK), p38 and other kinases [117, 118]. Specifically, activation of *p38* and *JUK* leads to inflammation and apoptosis, while ERK/MAPK signaling promotes cell growth and development, acting as anti-apoptotic signals. However, under some circumstances, ERK/MAPK can function in a pro-apoptotic manner [119]. For example, a previous study reported that inhibition of ERK expression protected neurons from low potassium conditions, while constitutive overexpression of ERK promoted cell death, suggesting an effect on neurodegeneration [120]. In our data, the expression of multiple components in all three of these pathways was significantly increased in SZ neurons, implying combinational effects towards apoptosis.

22q11.2 haploinsufficiency seems to have a robust and persistent impact on MAPK signaling pathway. As mentioned earlier, ERK/MAPK signaling was also found to be significantly disrupted in a transcriptomic study of peripheral blood mononuclear cells (PBMC's) from SZ patients with 22q11.2 deletion [69]. Knockout of *DGCR8*, which maps to the 22q11.2 deleted region, was reported to down-regulate ERK/MAPK and PI3K/AKT signaling in muscle [19]. Moreover, reduced expression of *ERK2* was reported in children with a 1 Mb micro-deletion in 22q11.2 [121]. Finally, abnormal activity of the MAPK signaling pathway was observed in postmortem SZ brain without 22q11.2 deletion [122, 123]. Taken together, these findings suggest that altered expression of MAPK signaling could be a common pathway in the pathogenesis of SZ.

We should, however, point out that abnormal apoptosis might occur in specific developmental period of SZ brains, rather than being a persistent event. While disrupted MAPK signaling was observed, no clear signs of aberrant apoptotic activity were detected in postmortem samples from SZ subjects. In fact, a reduction in apoptosis in brain autopsy samples and in fibroblasts treated with an apoptosis inducer has been found in SZ patients [124, 125], while increased susceptibility to apoptosis was reported in antipsychotic-naïve first-episode SZ patients [31].

By analyzing previously published three-dimensional chromatin interaction data, we found that in nuclei the 22q11.2 region could be physically juxtaposed to 6p21, where the *HLA* loci are located. Although this observation needs to be confirmed, it is consistent with previous reports that immunodeficiency is one of the key features of 22q11.2 DS [126, 127]. Interestingly, our pathway analysis of the differentially expressed genes also showed that immune response was among the most significant GO terms, after apoptosis and programmed cell death. However, as noted above, HLA proteins appear to have non-immune effects on synaptogenesis, which could also conceivably underlie potential functional associations between HLA and 22q11.2 DS [101–105].

To provide a perspective of how the DEGs from our in vitro study may be related to functional perturbations that can occur during brain development in SZ, we performed co-expression analysis of the DEGs using expression data from the BrainSpan. This analysis has been shown to be very enlightening for mapping specific genes and critical neurodevelopmental processes in time and space in the brain to clarify disease pathophysiology [64, 128]. Our analysis demonstrated that the DEGs, detected in 22q11.2 SZ neurons, converged on co-expression networks that could play critical roles in the development and function of specific brain regions in the embryonic and adolescence stages.

At the embryonic stage, *CDC45*, one of the five deleted genes known to modulate the cell cycle (the other four are *RANBP1, HTF9C, HIRA* and *UFD1L,* as noted above), showed the greatest connectivity with other DEGs, indicating that it may be the most impacted process. Analysis of such highly connected DEGs and the non-differentially expressed genes with high co-expression with the DEGs uncovered functional clusters involved in cell cycle, cell differentiation and cell growth. These results suggest that genes in these functional clusters may be co-regulated, and reduced dosages of 22q11.2 genes may disrupt the co-regulation relationship, compromising mitotic cell cycle regulation and neurogenesis in early embryonic brain development. Interesting, many of the 22q11.2 genes, including *CDC45*, are expressed at the highest level during mid to late gestation - the peak timing of neurogenesis - and then decrease substantially thereafter (Additional file 12) [71, 129], in support of their potential importance in early neurodevelopment.

Defects in cell proliferation and migration have been reported during cortical neurogenesis of *Lgdel* mice (the mouse model for the large 22q11.2 deletion) [71]. For instance, the proliferation of basal progenitors, a specific class of cortical precursor cells, was reported to be diminished in several regions of the embryonic *Lgdel* cortex, particularly anterior frontal regions [129]. Consequently, tangential migration of embryonic interneurons from the basal forebrain into the same cortical areas was also affected, since basal progenitors were unable to produce normal numbers of cortical projection neurons. In addition, altered cell cycle dynamics was seen in subsets of SZ-patients without 22q11.2 deletions [130, 131]. Our proliferation analysis of the 22q11.2 neural progenitor cells also supports these results.

Intracellular signaling pathways in the developing cortex also likely contribute to the progenitor regulatory network that includes 22q11.2 genes. It is known that the integrity of FGF signaling pathways is critical in the generation, proliferation and maintenance of neural progenitors [132, 133]. Distal deletion in the 22q11.2 region has been associated with basal progenitor defects via ERK/FGF signaling [121]. Therefore, we speculate that there may be a group of genes in the 22q11.2 region whose dosage is critical for intracellular signaling linked to cell proliferation. Indeed, function analysis of genes most co-expressed with the DEGs in embryonic brains fully supports this (Fig. 6). Also, several components (i.e. MAPK, APK3, FGFR2) from ERK/FGF signaling were among the top co-expressed DEGs in the embryonic stage (Table 3).

Noticeably, similar changes were not seen in *PRODH* mutant mice [134]. In our analysis, this gene, which encodes a key enzyme involved in proline catabolism, was instead found to have the largest number of connections to other DEGs, as well as to non-DEGs, in brain regions of the late adolescent period to early adulthood (Table 3). Function analysis of the co-expressed network in these late stage brain regions indicated that a subset of 22q11.2 genes may affect SZ brain development and act by disrupting mitochondrial function, particularly during activity-dependent synaptogenesis, which requires substantial metabolic support [135] (Fig. 7). This finding is consistent with a blood-based WGCNA analysis in 22q11DS patients in which a module of genes enriched for protein folding and metabolic process was identified to be associated with psychosis phenotype [57]. Interestingly, the network structure of this module could be re-established only in the adolescent stage of the BrainSpan data (WGCNA module preservation score > 2). Within the 22q11.2 region, there are six genes that are known to affect mitochondrial function: *PRODH, MRPL40, SLC25A1, TXNRD2, T10,* and *ZDHHC8*. Together they account for nearly 1/3 of the 22q11.2 genes expressed in brains [71]. The expression of these genes reaches their highest levels after birth (Additional file 12), supporting their essential roles in the late phase of neurodevelopment and neural function. It should be noted that a similar peak period of synapse formation occurs in the early postnatal primate brain, including humans. During this period, synaptic density reaches its maximum, followed by a progressive adjustment of synapses during adolescence [136, 137]. Hence, the diminished dosage of 22q11.2 genes that affect mitochondrial function, including *PRODH*, might further disrupt neural development by affecting metabolic/catabolic homeostasis during synapse formation.

Note that *PRODH* and *DGCR6¸*which map very closely to each other (25 kb), were the top two connecting deleted genes in the adolescence stage. Previous association analyses of various segments in the 22q11 deleted region using polymorphic markers for fine-scale mapping in heterogeneous U.S. family samples, determined that markers near *PRODH* and *DGCR6* were associated with SZ, providing strong evidence for a contribution of the *PRODH/DGCR6* locus in 22q11-associated SZ [14].

Finally, COMT (catechol-*O*-methyltransferase), which modulates cortical dopamine levels, was the third most connecting 22q11.2-linked gene, following *PRODH* and *DGCR6,* in the adolescence stages. Recently, epistatic interaction between the *PRODH* and *COMT* genes was demonstrated at the level of transcription, in which selective up-regulation of *COMT* in the prefrontal cortex was shown to respond to enhanced dopaminergic signaling in *PRODH*-deficient mice [138]. Thus, individuals with both SZ and 22q11.2 deletion may have an additive disadvantage because they have deficits in both genes and therefore might not be able to compensate for reduced *PRODH* expression, for the cortical dopaminergic hyperactivity caused by *PRODH* deficiency. Our results showed significant enrichment of protein catabolic/metabolic activities in the adolescence stages, but not in the embryonic stage, supporting the critical roles of *PRODH* and *COMT* in the later stages of neural development (Fig. 7; Additional file 11).

Despite these interesting findings and observations, there are a few caveats and limitations in our study that should be mentioned and need to be carefully overcome in future studies. First, we did not have a group of samples from 22q11.2 carriers without a SZ or SAD diagnosis. An inclusion of them may help us to disentangle the factors that depend only on the 22q11.2 deletions but do not necessarily contribute to SZ pathogenesis directly. Secondly, the heterogeneity between samples was relatively large, even in those from the same individuals. Here we applied advanced analytic approaches to reduce this confounder, but it will be much better to generate more samples and then select the homogenous ones for gene expression comparison. With an increased number of samples, we will also be able to apply FDR rather than nominal *p*-values to select DEGs for the downstream analysis, which will enhance the validity of our findings. Thirdly, it will be valuable to perform similar systematic gene expression analyses using samples at different stages of neuronal differentiation, including mature neurons.

## Conclusions

Gene expression profiling using neurons derived from iPSCs, and our bioinformatics analysis, have provided evidence for potential molecular connections between 22q11.2 deletion and SZ brain development, and a rationale for studying potentially druggable targets, such as MAPK, in treating the psychiatric manifestations of neuropsychiatric disorders associated with 22q11.2 del.

## Additional files

Additional file 1: Table S1. Is a table listing the patients and controls used in this study. (XLSX 37 kb)
Additional file 2: Text S1. Is a text file including expanded clinical details of subjects used for iPSC development, additional experimental methods and materials. (DOCX 630 kb)
Additional file 3: Figure S1. Is a figure showing sample clustering and “batch” correction. A) UPGMA clustering of samples based on expression of all transcripts with FPKMs ≥1. Similarities of transcriptomic profiles between samples were determined using Pearson Correlation. B) Heat map showing relative expression of 55 known neural stem cell and differentiating neuronal markers. (TIF 525 kb)
Additional file 4: Figure S2. Is a figure to demonstrate neural fate and maturity of our samples. *A*) Non-metric multidimensional scaling on 22q11DEL samples and fetal/adult human cortical neurons for the first two dimensions. B) PCA on 22q11DEL samples and temporal dataset of human cortical neurons for the two three principal components. C) Heat map showing relative expression of markers genes to demonstrate the relative yield of glutamatergic, GABAergic neurons and astrocytes. (PDF 986 kb)
Additional file 5: Table S2. Is a table listing summary statistics of RNA-seq. (XLSX 11 kb)
Additional file 6: Table S3. Is a table listing the entire gene list with corrected expression values. (XLS 5575 kb)
Additional file 7: Table S4. Is a table listing the up-regulated genes in SZ. (XLS 212 kb)
Additional file 8: Table S5. Is a table listing the down-regulated genes in SZ. (XLS 127 kb)
Additional file 9: Figure S3. Is a figure with a bar plot presenting uncorrected expression values of 22q11.2 genes in control and SZ samples. Three genes flanking the deleted region at either side were also included. Asterisks (*) on the top of genes indicated significantly differential expression at the genome-wide scale (FDR < 0.05). (XLS 530 kb)
Additional file 10: Figure S4. Is a figure with a histogram illustrating distribution of number of connections per gene in the FC1. A small number of DEGs account for the majority of the connections in the networks. (TIF 43 kb)
Additional file 11: Table S6. Is a table listing enriched GO terms of the co-expression networks in different regions of the embryonic and adolescence brains. (XLS 482 kb)
Additional file 12: Figure S5. Is a figure with a heat map showing relative expression of 22q11 DEL genes in BrainSpan data in the frontal cortex. Red color indicates higher expression value, while blue means lower expression. (TIF 339 kb)

## Abbreviations

22q11.2 DS: 22q11.2 Deletion Syndrome
ASD: Autism spectrum disorders
DEGs: Differentially expressed genes
FDR: False discovery rate
FPKM: Fragments per kilobase of exon per million fragments mapped
GWAS: Genome wide association studies
iPSCs: Induced pluripotent stem cells
lincRNAs: Long intergenic non-coding RNAs
NPC: Neural progenitor cells
RNA-seq: RNA deep sequencing
SAD: Schizoaffective disorder
SZ: Schizophrenia
VCFS: Velo-cardio-facial syndrome
WGCNA: Weighted Gene Coexpression Network Analysis
