# Supplementary material for: Integrative transcriptome network analysis of iPSC-derived neurons from schizophrenia and schizoaffective disorder patients with 22q11.2 deletion
Source: BMC Syst Biol. 2016 Nov 15;10:105. doi: 10.1186/s12918-016-0366-0 (PMC5111260; doi:10.1186/s12918-016-0366-0)
Supplement: Additional file 2: Text S1. — Is a text file including expanded clinical details of subjects used for iPSC development, additional experimental methods and materials. (DOCX 630 kb) [file 12918_2016_366_MOESM2_ESM.docx]

Integrative Transcriptome Network Analysis of iPSC-derived Neurons from Schizophrenia and Schizoaffective Disorder Patients with 22q11.2 Deletion

Mingyan Lin^1^, Erika Pedrosa^2^, Anastasia Hrabovsky^2^, Jian Chen^2^, Benjamin R. Puliafito^2^, Stephanie R Gilbert^1^, Deyou Zheng^1,3,4, *^, Herbert M. Lachman^1,2,4,5,*^

1 Department of Genetics, 2 Department of Psychiatry and Behavioral Sciences, 3 Department of Neurology, 4 Department of Neuroscience, 5 Department of Medicine, Albert Einstein College of Medicine, 1300 Morris Park Ave., Bronx, New York USA

*co-corresponding authors

Herbert Lachman, MD

Department of Psychiatry, Albert Einstein College of Medicine

1300 Morris Park Ave., Bronx, New York 10461

Phone:718-430-2428; email: herb.lachman@einstein.yu.edu

Deyou Zheng, Ph.D.

Department of Neurology, Albert Einstein College of Medicine

1300 Morris Park Ave., Bronx, New York 10461

Phone: 718-678-1217; email: deyou.zheng@einstein.yu.edu

**Supporting Information**

**I. Supplemental Methods. Expanded clinical details of subjects used for iPSC development and experimental methods**

**Subjects used for iPSC development.** The controls ctrl_iPSC1, ctrl_iPSC2, ctrl_iPSC5, ctrl_iPSC6, and the patients SZ_iPSC15 SZ_22q11-30 SZ_22q11-10 SZ_22q11-60, were recruited at the Albert Einstein College of Medicine. The controls ctrl_553 and ctrl_690, and the patients SZ_1804 SZ_1220, were ascertained at the NIMH, Child Psychiatry Branch, by Dr. Judy Rapoport, as part of her ongoing childhood onset schizophrenia study(1, 2). Fibroblasts were sent by the group for iPSC development. The Einstein subjects were diagnosed with VCFS based on typical physical manifestations; the diagnosis was confirmed by FISH. Psychiatric diagnoses were established many years prior to recruitment by the patient’s psychiatrists using non-structured clinical interviews. A history of psychosis was confirmed by non-structured clinical interview with the patients and a parent. Subject SZ_22q11-10 had congenital heart disease (VSD) and cleft palate. There was a history of mild developmental delay with the first episode of psychosis occurring at age 14 characterized by excitability and grandiosity. This was followed by the development of paranoia and auditory hallucinations. Subject SZ_ iPSC-15 had typical VCFS with cleft palate, an extra digit, and a single kidney. He had pharyngeal flap surgery. The patient was diagnosed with major depression at age 16 and experienced severe temper tantrums. At age 30 he developed auditory hallucinations that lasted for ~ 1 year, which was controlled with perphenazine. There was no history of grandiosity, pressured speech, or flight of ideas. Patient SZ_22q11-30 was diagnosed with VCFS as a child. She had a VSD repaired at age 7, and pharyngeal flap surgery at ages 5 and 9 to correct severe speech impediment. At age 37, she became delusional, believing that she had physically injured her parents. She also displayed incoherent speech and became depressed. No history of paranoia or persecution, but delusion about hurting parents persists. She has also experienced the delusional belief that the person she sees on TV or in magazines is herself; she now avoids these activities because the fear these delusions elicit. There have been several admissions in the past year for suicidal ideation. Patient SZ_22q11-60 has VCFS with pulmonary stenosis as the major physical manifestation. There is a long history of paranoia, depression, and grandiosity.

Patients with childhood onset schizophrenia (COS), recruited as part of an NIMH initiative, met DSM-IIIR/DSM-IV criteria for SZ with documented onset of psychosis before age 13 were recruited. They were subsequently interviewed for lifetime and current psychiatric disorders using structured psychiatric interviews, with diagnosis confirmed by inpatient, medication-free observation(1, 2).

Controls in both cohorts were assessed by non-structured clinical interviews. There was no personal history of an Axis I diagnosis, and they have never been treated for a psychiatric disorder.

**Harvesting Fibroblasts.** Skin biopsies were performed in consenting individuals by a board-certified dermatologist. Skin biopsy samples were transferred to a small Petri dish containing 2-3 ml Skin Fibroblast Media (SFM) consisting of RPMI 1640, 10% FBS, 1% pen/strep, 10ng/ml FGF2. The sample was incubated at room temperature for 15 minutes. Medium was carefully aspirated and replaced with 1-2 ml of collagenase type II solution (3mg/ml collagenase II dissolved in DMEM High Glucose [Worthington Biochemical Corp. Lakewood, NJ: GIBCO/Invitrogen, Carlsbad, CA]). The tissue was chopped into small pieces using 2 sterile scalpels, after which they were allowed to incubate at 37^o^C for 1­2 hours depending on size. The sample was then collected in a 15ml falcon tube and washed with SFM (serum free medium). Tissue was collected by centrifugation at 100G for 4 minutes. The pelleted sample was then suspended in SFM and plated in a T12.5 ml flask at 37^o^C in 5% CO_2_ for 3 days without changing medium or manipulation, to allow fibroblasts to adhere. Then cells were subsequently fed every 2 days with RPMI 1640 containing 10% FBS until a confluent culture was obtained (~3 weeks). The cells were reprogrammed into iPSCs as described in the main methods section of the paper.

**Establishing Human iPSCs.**  iPSC reprogramming was carried out by nucleofection. One vial of cells was thawed out and placed in a T75 flask in DMEM/F12 supplemented with 10% FBS and fed every 2 days. Cells were grown to ~50% confluence (~4-5 days), after which they were trypsinized and subjected to nucleofection (~6 x105 cells). Reprogramming was carried out using an Amaxa 4D-Nucleofector (P2 Primary Cell Kit from Lonza cat# V4XP-2012, Program FF-135) with non-integrating plasmids containing OCT4, SOX2, KLF4, L-MYC, LIN28, and a p53 shRNA vector (Addgene Cat. # 27077, 27078, 27080), according to Okita et al., with some modifications(3-5). iPSCs were maintained on Matrigel plates in mTeSR1 medium (Stem Cell Technologies) with daily feeding in 37^o^C/5% CO_2_/85% humidity.

**Germ line markers, establishing pluripotency by *in vitro* differentiation and karyotype.** Pluripotency for all iPSC lines was confirmed by immunocytochemistry using antibodies (Ab) against Tra-1-60, Tra-1-81, SSEA3 and SSEA4, which are expressed in pluripotent stem cells. In addition, the capacity to differentiate into all 3 germ layers was established by *in vitro* assays, as previously described(4-6). The markers desmin (mesoderm), α-fetoprotein (endoderm), and βIII-tubulin (ectoderm) were used(7-10). A list of the Ab used in the study is shown below. Karyotyping was carried out by Cell Line Genetics (Madison WI). All lines had normal karyotypes**.**

**Neuronal differentiation.** iPSCs were maintained in mTeSR1 medium (Stem Cell Technologies) for approximately 5-6 days. Colonies were checked for spontaneous differentiation under a dissecting microscope; clusters of differentiated cells were removed manually. The medium was then changed to N2 (DMEM/F12, 1X N2; Invitrogen). After 24 hours, medium was changed to N2 plus 1μM Dorsomorphin (CALBIOCHEM). The following day, embryoid bodies (EBs) were created. Briefly, iPSCs were checked again for spontaneous differentiation and fresh N2 medium plus 1μM Dorsomorphin was added. Colonies were cut with a 5ml glass serological pipet using wide strokes to generate large fragments. A cell scraper was then used to detach remaining cells. EBs were aliquoted to a 6-well, ultra-low attachment plate (Corning). Two days later, EBs were collected in a 15ml tube and allowed to settle by gravity for 5 minutes. Supernatant was removed and fresh N2 media plus 1μM Dorsomorphin was added. EBs were aliquoted to a new ultra-low attachment plate. From this point, EBs were fed every other day for 6 days, after which neuronal differentiation was induced. NPCs were generated from neural rosettes as previously described by Marchetto et al. with slight modifications *vitro(4, 11, 12)*. Briefly, EBs were collected in a 15ml tube and allowed to settle by gravity for 5 minutes. Supernatant was removed and EBs were gently resuspended in NBF medium (DMEM/F12, 0.5X N2, 0.5X B27, 1% p/s) plus fresh 20ng/ml FGF2 (R&D Systems). EBs were gently aliquoted to a matrigel (BD Biosciences) plate using a 10 ml pipet. Two days later, plates were checked for rosette formation and fed with NBF medium plus fresh FGF2. Rosettes were fed every other day x 2, then carefully excised with a 26g needle and pooled in a 1.5ml tube. Accutase (ICT) was added to the rosettes for 3 minutes at 37^o^C. After incubation, rosettes were broken up with a 1ml pipet tip, centrifuged for 2 minutes at 100G, and washed once with 1X PBS (Invitrogen). The pellet was resuspended as single cells in NBF media with fresh FGF2 (20ng/ml) and aliquoted onto Poly-L-Ornithine (Sigma)/Laminin (Roche) plates. NPCs were fed every other day. Once NPCs reached ~50% confluence, neural differentiation was initiated by withdrawing FGF2 and adding NBF media supplemented with fresh growth factors as follows: WNT3A (100ng/ml) (R&D Systems), BDNF (10ng/ml), GDNF (10ng/ml), IGF-1 (10ng/ml) (PeproTech), and cAMP 1μM (Sigma). Cells were fed every other day. The
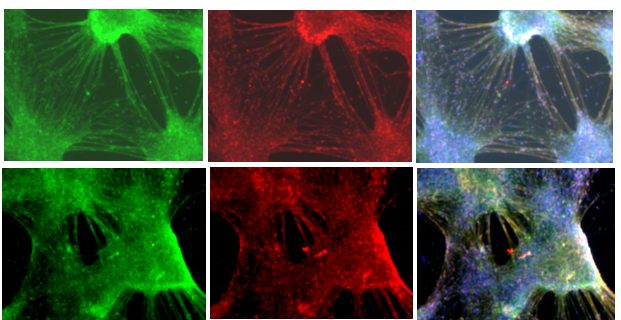
protocol produces a heterogeneous mix of glutamatergic neurons and GABAergic neurons (about 50 to 50), often with co-staining in the same cells.

Immunohistochemistry of day 14 control neurons: vGLUT2 (left); GAD65/67 (middle); Merge (right)


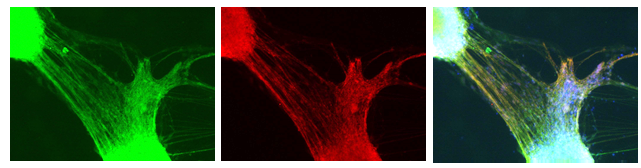


Immunohistochemistry of day 14 neurons, 22q11.2 del: vGLUT2 (left); GABA (middle); Merge (right)

**Reverse transcribed PCR (RT-PCR) and quantitative real-time PCR (qPCR)**

Total RNA was extracted using a miRNeasy Kit according to the manufacturer’s instructions (Qiagen). An additional treatment with DNase1 (Qiagen, Valencia, CA) was included to remove genomic DNA. Reverse transcribed PCR (RT-PCR) was performed using a OneStep RT-PCR Kit (Qiagen, Valencia, CA) according to the manufacturer’s instructions. The cDNA was used as a template for quantitative PCR (qPCR), which was carried out using the ABI 7900HT Real-Time PCR System (Applied Biosystems, Foster City, CA). Each reaction consisted of cDNA, primers, and SYBR Green PCR Master Mix (Applied Biosystems, Foster City, CA) in an 8 μl volume. Melting curve analysis of target sequences showed that all primers used in this study generated amplicons that had a single peak, without primer-dimer artifacts. Primer concentrations were optimized prior to use in qPCR experiments. Relative changes in gene expression were calculated using the 2^-∆∆Ct^ method with β2-microglobulin (β2M) as a reference gene. Each qPCR was carried out in triplicate, with each triplicate data point repeated 3 times. For the triplicates, only samples that differed by <0.3 Ct values were used in the final calculations. Less than ~5% of samples fell out of this range. In addition, standard curves were generated for each gene using a 50-fold dilution range. qPCR experiments were only used in the final analysis if the slope of the Ct vs input curve was at least -3.0 and the correlation coefficient for triplicate sames was >0.98. Relative changes in gene expression were calculated using the 2^-∆∆Ct^ method with β2-microglobulin (β2M) as a reference gene. Significant differences in gene expression were assessed using a two-tailed student T-test.

**PCR primers used in this study**

**Gene Forward Reverse**

OCT4 plasmid CATTCAAACTGAGGTAAGGG TAGCGTAAAAGGAGCAACATAG

KLF4 plasmid CCACCTCGCCTTACACATGAAGA GCGTAAAAGGAGCAACATAG

SOX2 plasmid TTCACATGTCCCAGCACTACCAGA TTGTTTGACAGGAGCGACGAT

L-MYC plasmid GGCTGAGAAGAGGATGGCTAC TTTGTTTGACAGGAGCGACGAT

LIN28 plasmid AGCCATATGGTAGCCTCATGTCCGC TAGCGTAAAAGGAGCAACATAG

β2M GCTCGCGCTACTCTCTCTTT CAATGTCGGATGGATGAAAC

DGCR8 CTCACCCACCCTCTGTGTTT CTTGATGGTTTGCCTCCAAT

IFITM3 CTGATTCTGGGCATCCTCAT ATACAGGTCATGGGCAGAGC

SSTR2 CTTTGTGGTGGTCCTCACCT GCAGAGGACATTCTGGAAGC

SLC25A1 TGTGGCTGTCAAATCCATGT CACAGGGTCATAGGCCAGAT

MAP3K7 GTTCCGTGTAAGGGCTTTGA GAGCAGCTGCCACTTACCTT

**Antibodies used in this study**

| **Antibody** | **Company** | **Catalog #** |
| --- | --- | --- |
| Anti-human Tra 1-60 | eBioscience | 12-8863-80 |
| Anti-human Tra 1-81 | eBioscience | 12-8883-80 |
| AF488 Anti-mouse/human SSEA-3 | eBioscience | 53-8833-71 |
| AF488 Mouse anti SSEA-4 | BD Pharmingen | 560308 |
| Anti-Tubulin, beta III isoform | Millipore | MAB1637 |
| Desmin Ab-1 | ThermoScientific | MS-376-S |
| Anti-human/mouseα-Fetoprotein | R & D | MAB1368 |
| PSD95 (mouse) | UC Davis/NIH NeuroMab Facility | 75-028 |
| Synaptophysin(rabbit) | Abcam | ab8049 |
| Anti-GAD65/67 | Sigma | G5163 |
| Ms anti- Vglut2 | Millipore | MAB5504 |
| Rabbit neuronal class III β-tubulin | Fisher | NC9168644 |
| Tbr1 | Abcam | Ab31940 |
| Sheep anti-Tyrosine Hydroxylase | Pel-Freez | P60101 |

Table S1. Demographics of subjects used for generating iPSCs

| Sample ID | Age at biopsy/sex | Diagnosis |
| --- | --- | --- |
| ctrl_iPSC1 | 29/F | control |
| ctrl_iPSC2 | 58/M | control |
| ctrl_iPSC5 | 32/M | control |
| ctrl_iPSC6 | 46/M | control |
| ctrl_553 | 31/M | control |
| ctrl_3113 | 18/F | control |
| ctrl_690 | 27/M | control |
|  |  |  |
| SZ_iPSC15 | 31M | SAD/VCFS |
| SZ_22q11-30 | 41/F | SZ/VCFS |
| SZ_537 | 34/M | COS |
| SZ_1804 | 25/F | COS |
| SZ_1220 | 31/F | COS |
| SZ_22q11-10 | 37/M | SAD/VCFS |
| SZ_22q11-60 | 25/M | SAD/VCFS |
| SZ_1275 | 28/F | COS |

References

1. Ahn K*, et al.* (2014) High rate of disease-related copy number variations in childhood onset schizophrenia. *Molecular psychiatry* 19(5):568-572.

2. Ahn K, An S, Shugart Y, & Rapoport J (2014) Common polygenic variation and risk for childhood-onset schizophrenia. *Molecular psychiatry*.

3. Okita K*, et al.* (2011) A more efficient method to generate integration-free human iPS cells. *Nature methods* 8(5):409-412.

4. Lin M*, et al.* (2011) RNA-Seq of human neurons derived from iPS cells reveals candidate long non-coding RNAs involved in neurogenesis and neuropsychiatric disorders. *PLoS One* 6(9):e23356.

5. Chen J*, et al.* (2013) Transcriptome comparison of human neurons generated using induced pluripotent stem cells derived from dental pulp and skin fibroblasts. *PloS one* 8(10):e75682.

6. Pedrosa E*, et al.* (2011) Development of patient-specific neurons in schizophrenia using induced pluripotent stem cells. *Journal of neurogenetics* 25(3):88-103.

7. Muenthaisong S*, et al.* (2012) Generation of mouse induced pluripotent stem cells from different genetic backgrounds using Sleeping beauty transposon mediated gene transfer. *Experimental cell research* 318(19):2482-2489.

8. Pal R, Mamidi MK, Das AK, & Bhonde R (2013) Comparative analysis of cardiomyocyte differentiation from human embryonic stem cells under 3-D and 2-D culture conditions. *Journal of bioscience and bioengineering* 115(2):200-206.

9. Takahashi K & Yamanaka S (2006) Induction of pluripotent stem cells from mouse embryonic and adult fibroblast cultures by defined factors. *cell* 126(4):663-676.

10. Takahashi K*, et al.* (2007) Induction of pluripotent stem cells from adult human fibroblasts by defined factors. *cell* 131(5):861-872.

11. Marchetto MC*, et al.* (2010) A model for neural development and treatment of Rett syndrome using human induced pluripotent stem cells. *Cell* 143(4):527-539.

12. Lin M*, et al.* (2012) Allele-biased expression in differentiating human neurons: implications for neuropsychiatric disorders. *PLoS One* 7(8):e44017.
