# Supplementary figures and images for: Integrative transcriptome network analysis of iPSC-derived neurons from schizophrenia and schizoaffective disorder patients with 22q11.2 deletion
Source: BMC Syst Biol. 2016 Nov 15;10:105. doi: 10.1186/s12918-016-0366-0 (PMC5111260; doi:10.1186/s12918-016-0366-0)

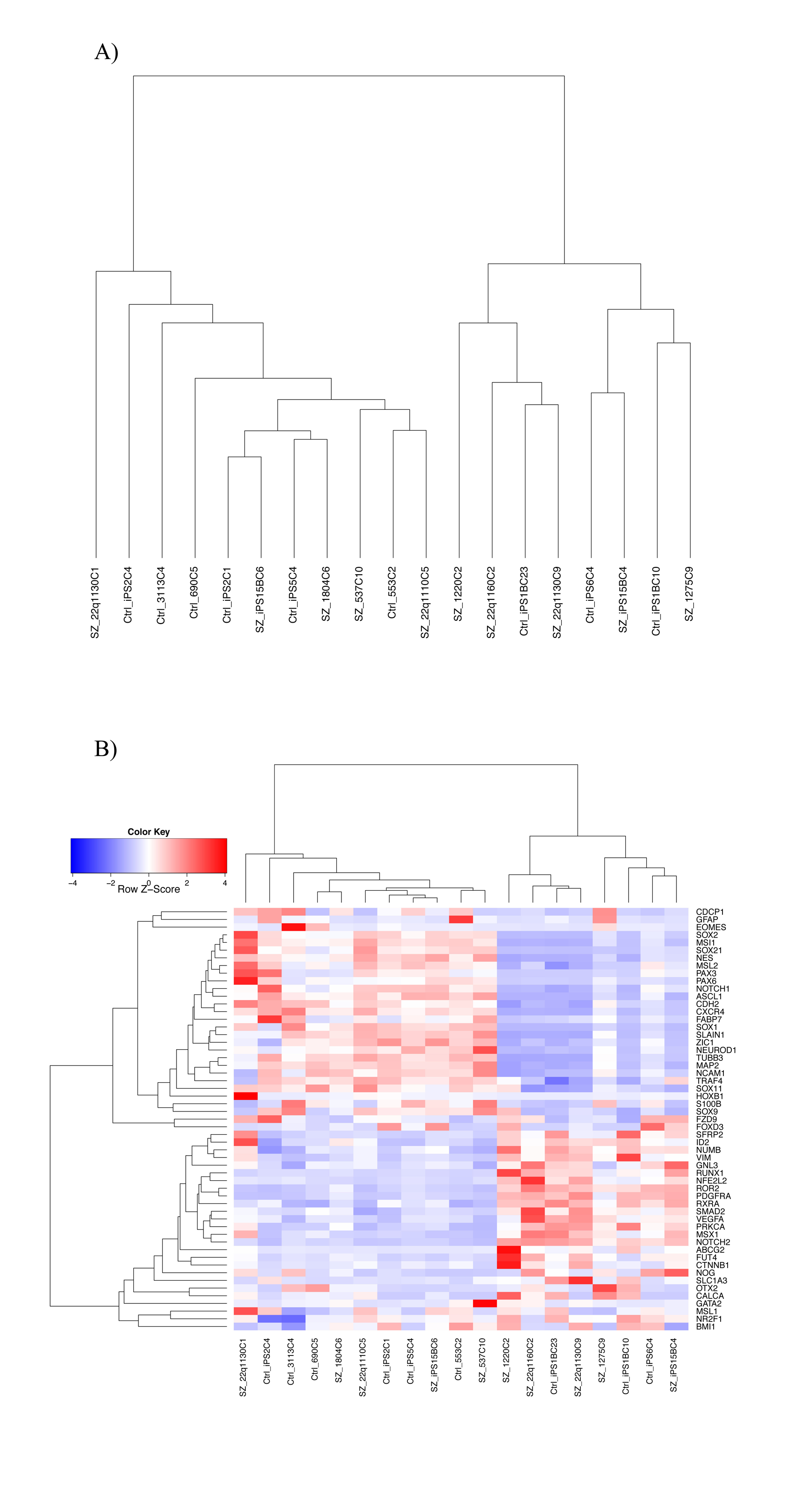

Supplement: Additional file 3: Figure S1. — Is a figure showing sample clustering and “batch” correction. A) UPGMA clustering of samples based on expression of all transcripts with FPKMs ≥1. Similarities of transcriptomic profiles between samples were determined using Pearson Correlation. B) Heat map showing relative expression of 55 known neural stem cell and differentiating neuronal markers. (TIF 525 kb) [file 12918_2016_366_MOESM3_ESM.tif]

A)

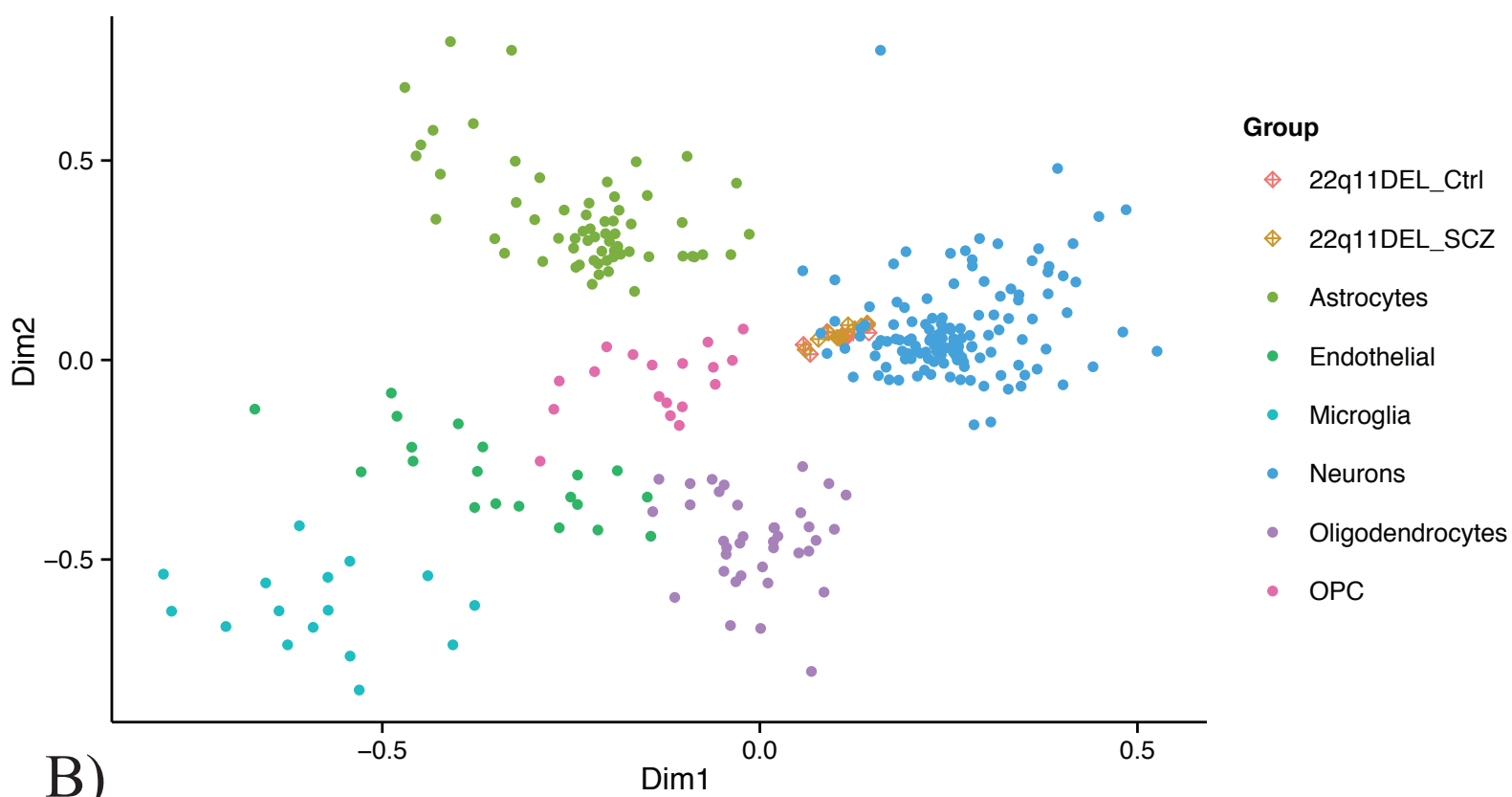

B)

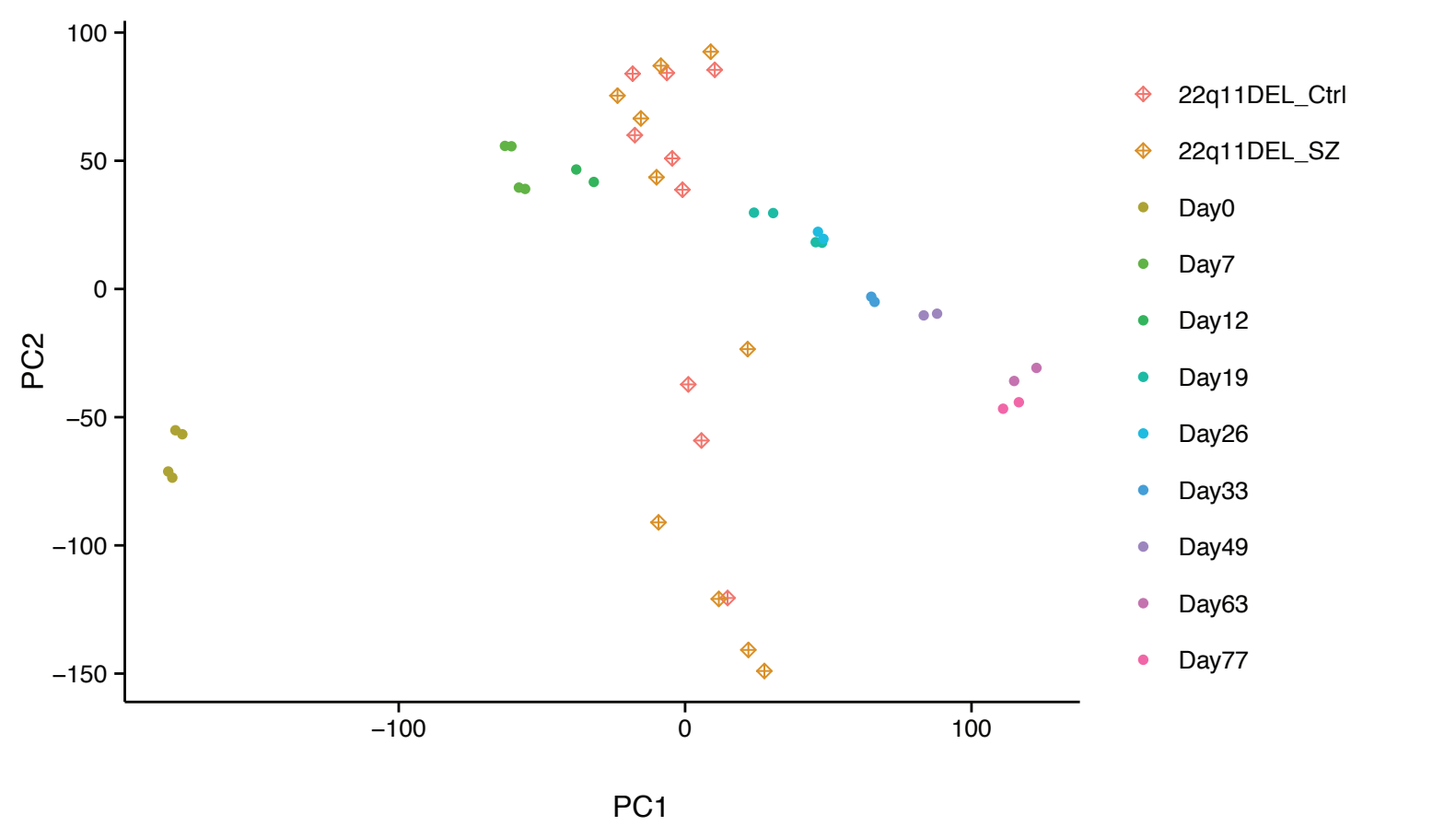

c)

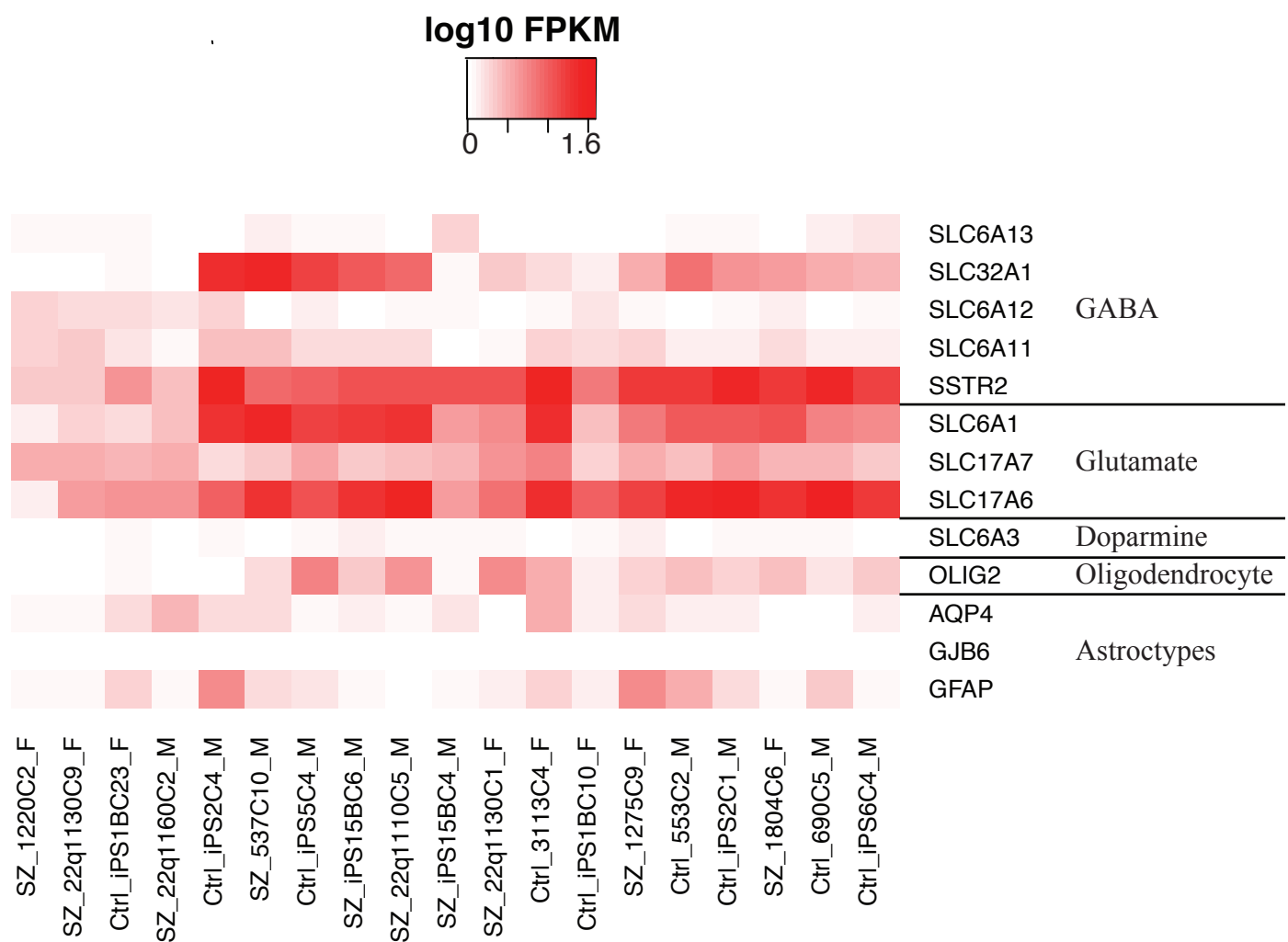

Supplement: Additional file 4: Figure S2. — Is a figure to demonstrate neural fate and maturity of our samples. A) Non-metric multidimensional scaling on 22q11DEL samples and fetal/adult human cortical neurons for the first two dimensions. B) PCA on 22q11DEL samples and temporal dataset of human cortical neurons for the two three principal components. C) Heat map showing relative expression of markers genes to demonstrate the relative yield of glutamatergic, GABAergic neurons and astrocytes. (PDF 986 kb) [file 12918_2016_366_MOESM4_ESM.pdf]

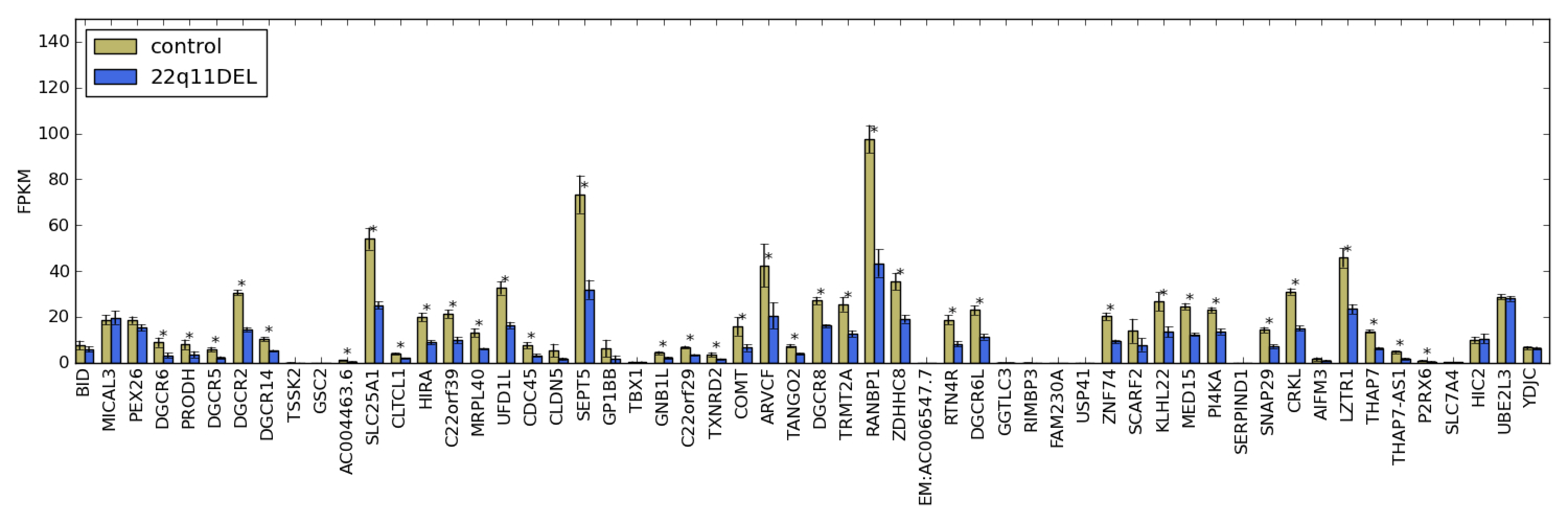

Supplement: Additional file 9: Figure S3. — Is a figure with a bar plot presenting uncorrected expression values of 22q11.2 genes in control and SZ samples. Three genes flanking the deleted region at either side were also included. Asterisks (*) on the top of genes indicated significantly differential expression at the genome-wide scale (FDR < 0.05). (XLS 530 kb) [file 12918_2016_366_MOESM9_ESM.xls]

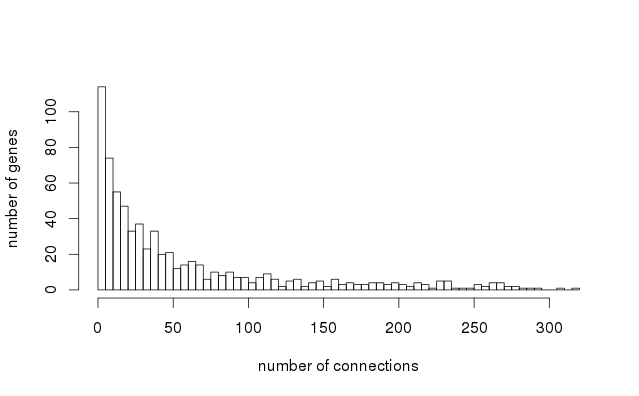

Supplement: Additional file 10: Figure S4. — Is a figure with a histogram illustrating distribution of number of connections per gene in the FC1. A small number of DEGs account for the majority of the connections in the networks. (TIF 43 kb) [file 12918_2016_366_MOESM10_ESM.tif]

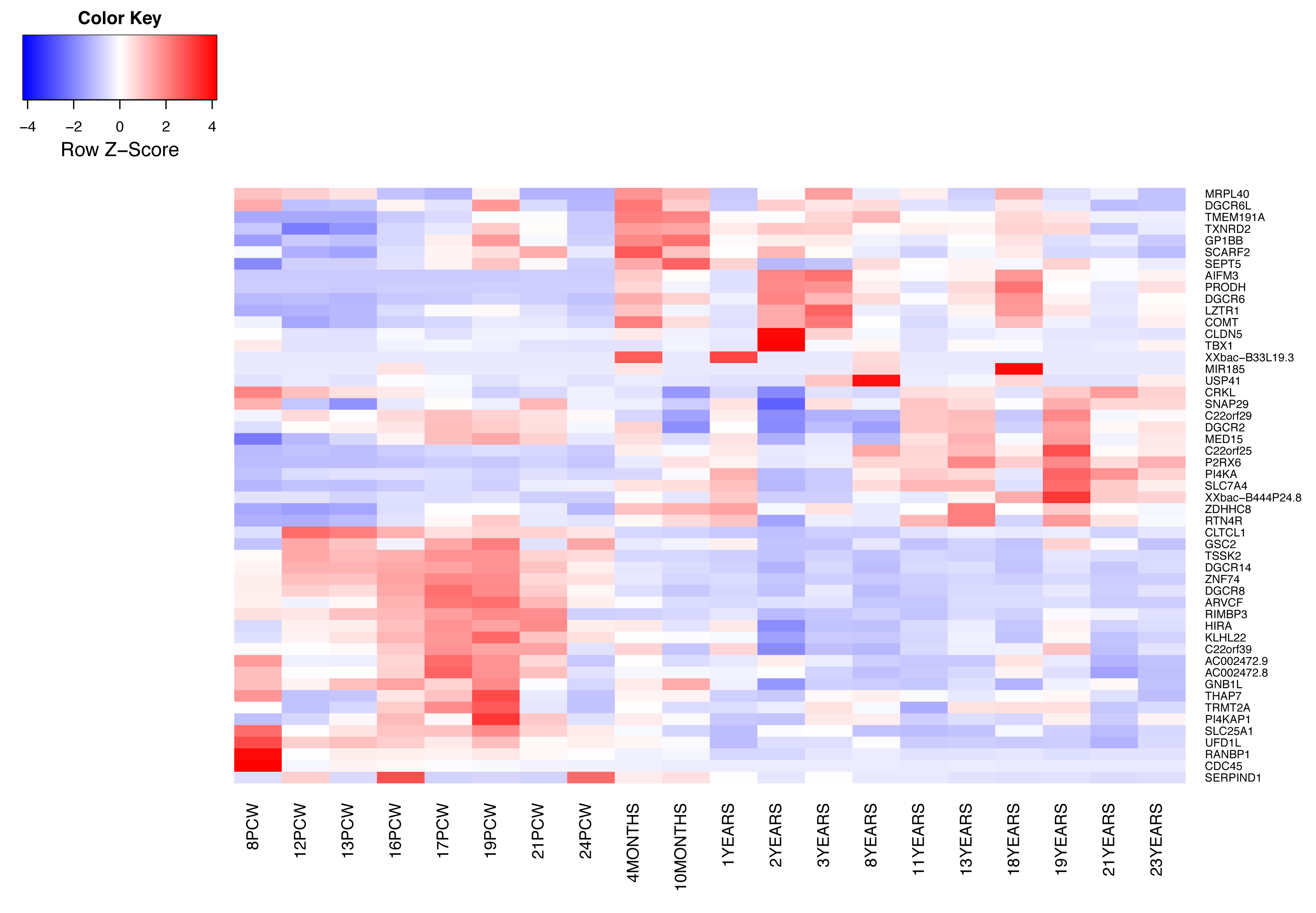

Supplement: Additional file 12: Figure S5. — Is a figure with a heat map showing relative expression of 22q11 DEL genes in BrainSpan data in the frontal cortex. Red color indicates higher expression value, while blue means lower expression. (TIF 339 kb) [file 12918_2016_366_MOESM12_ESM.tif]
